# The effects of *Pinus sylvestris* L. geographical origin on the community and co-occurrence of fungal and bacterial endophytes in a common garden experiment

**DOI:** 10.1128/spectrum.00807-24

**Published:** 2024-09-09

**Authors:** Pulak Maitra, Katarzyna Hrynkiewicz, Agnieszka Szuba, Adrianna Niestrawska, Joanna Mucha

**Affiliations:** 1 Institute of Dendrology, Polish Academy of Sciences, Kórnik, Poland; 2 W.K. Kellogg Biological Station, Michigan State University, Hickory Corners, Michigan, USA; 3 Department of Microbiology, Faculty of Biological and Veterinary Sciences, Nicolaus Copernicus University in Toruń, Toruń, Poland; Oulun yliopisto, Oulu, Finland

**Keywords:** scot pine, root microbial communities, tree origin, common-garden, season

## Abstract

Below-ground microorganisms, particularly endophytes, are pivotal for plant establishment and functioning through nutrient acquisition and enhancing resistance to abiotic and biotic stresses. The impact of host plant origin within a species on the composition and interaction networks of root endophytic fungi and bacteria has been less explored compared with plant phylogeny and biological distance. This study investigates the effect of geographic origin on the fungal and bacterial microbiomes of *Pinus sylvestris* L. root endophytes. Roots from plants grown in a common garden, originating from six locations, were harvested in two distinct seasons. Fungal and bacterial microbiomes were analyzed using Illumina MiSeq sequencing. The operational taxonomic unit (OTU) richness of endophytic fungi and bacteria showed no significant variation due to tree origin or season. However, the Shannon diversity index for endophytic fungi was seasonally influenced. The composition of endophytic fungal and bacterial communities was affected by both tree origin and season, correlating with host root biochemical parameters, such as starch, total non-structural carbohydrates, carbon, nitrogen, and climatic factors, such as mean annual precipitation and temperature. Moreover, the abundance of specific endophytic fungi and bacteria varied across different *P. sylvestris* origins, depending on the season. The complexity of the co-occurrence networks of fungal and bacterial endophytes within *P. sylvestris* also differed by geographical origin and season. This study highlights the significant role of biochemical and climatic factors associated with tree origin in shaping interactions with endophytic communities, potentially affecting plant health and adaptability across diverse environments.

**IMPORTANCE:**

This study advances our understanding of how plant ecotype and seasonal changes influence root endophytic communities in Scots pine (*Pinus sylvestris*). By examining trees from various origins grown in a common garden, it highlights the role of tree origin and season in shaping fungal and bacterial community and co-occurrence networks. Importantly, this research demonstrates that tree origin impacts the composition and interaction networks of root endophytes and depends on the season. The study's findings suggest that root biochemical traits and climatic conditions (e.g., temperature, precipitation) associated with tree origin are crucial in determining the assembly of endophytic communities. This understanding could lead to innovative strategies for enhancing plant health and adaptability across different environments, contributing to forestry and conservation efforts. The research underscores the complexity of plant–microbe interactions and the need for a comprehensive approach to studying them, highlighting the interplay between tree origin and microbial ecology in forest ecosystems.

## INTRODUCTION

The below-ground microbial community, particularly the endophytic community, plays a crucial role in forest ecosystems' establishment and maintenance. Endophytic fungi and bacteria aid plants in overcoming abiotic and biotic challenges, such as nutrient and water scarcity, temperature extremes, and pathogen infections (1–6). Endophytes are particularly vital during the early growth stages of tree seedlings, aiding adaptation in new locations with adverse soil or climatic conditions and positively influencing seedling survival and vegetation dynamics (7, 8). However, soil-resident endophytic microbial taxa may colonize new trees, exhibiting preferences for specific hosts, whereas plant roots may selectively filter beneficial microorganisms (9, 10). Fungal and bacterial endophytes can directly or indirectly affect plant biochemical properties, influencing tolerance to unfavorable climatic conditions. Thus, the ability of plant species to thrive under climate change may be intricately linked to the diversity, composition, and interactions of their endophytic root communities.

Variability in fungal and bacterial root communities is influenced by host plant phylogeny and taxonomic distance (11, 12), but the impact of host plant origin within a species on root endophytes is less understood (13, 14). Soil properties significantly shape root–microorganism interactions (15), whereas root traits, modulated by climatic factors, such as temperature (16) and precipitation (17), influence carbon allocation patterns within plant ecotypes. Despite uniform resource availability in common gardens, phenotypic variations arise from plant genotypes or ecotypes, affecting microorganism colonization (18). Such root phenotypic trait variations within a species, including carbon and nitrogen content, significantly impact their interacting microorganisms. Previous studies have shown that plant phenotypes, influenced by both genetic and environmental factors, significantly affect phyllosphere and endophytic fungal and bacterial communities (19, 20). This study is novel in examining the impact of plant geographical origin on endophytic bacterial communities, expanding on research exploring the effects of plant genotype and geographical origin/ecotypes on root fungal communities in specific species (14, 21). Significant differences in root fungal communities across ecological types, phenologies, and genotypes with varying nutrient and drought resistance have been noted (22–25). In *Populus angustifolia*, genotype variations led to distinct ectomycorrhizal fungal compositions (21), associated with plant traits, such as structural compounds and nutrient mineralization (26–28). This interaction may be modulated by seasonal abiotic changes, such as temperature and moisture, which cause metabolic shifts in plants, thus affecting the dynamics of microbial communities (29, 30). Seasonal fluctuations in plant compounds, such as sugars, proteins, amino acids, and organic acids, significantly impact associated microbial communities (31–33). For example, fungal diversity, community composition, and abundance have been observed to vary significantly across growing seasons (29, 30, 34, 35). However, the impact of geographical origin on root endophytic communities across different growing season, especially bacterial ones, remains underexplored, particularly in adult trees.

Ecological network analysis of coexisting microbial species elucidates the principles underlying plant species coexistence, community formation, and stability (36–38). Comparative analyses of microbiomes indicate that microbial groups, sensitive to environmental factors, exhibit distinct network characteristics (36, 39–42). Management practices and tillage intensity are known to affect cross-kingdom co-occurrence networks (39). Previous research has shown that tree genotype and growth stage impact rhizosphere bacterial network structures (43), rhizosphere bacterial networks in cotton are influenced by host genotypes (44), and phyllosphere endophytic fungal–bacterial networks in wild *Arabidopsis thaliana* are shaped by host genotypes and growing seasons (42, 45). However, the influence of *P. sylvestris* trees' geographical origin/ecotype on the co-occurrence of endophytic fungal and bacterial communities remains unexplored, particularly in the context of long-lived trees.

A wide range of habitats has led to the development of numerous ecotypes, morphotypes, and even varieties within *P. sylvestris* trees (46). Provenance trials and common garden experiments have documented significant ecotypic heterogeneity in growth features (47, 48). This study aims to examine the influence of tree origin (ecotype) on the diversity, composition, and co-occurrence of root endophytic fungal and bacterial communities in translocated Scots pine (*P. sylvestris*) in Poland across two growing seasons (spring and autumn). Roots from 60 individual *P. sylvestris* trees, representing six different geographical origins of seeds, were harvested in both seasons from a common garden site established approximately 50 years ago. Common garden experiments facilitate the investigation of ecotypic variation by controlling for confounding environmental factors (e.g., temperature, precipitation, soil parameters) (14). The composition of Scots pine root-associated fungal and bacterial communities was analyzed using Illumina MiSeq sequencing. This study investigates whether ecotypic variants of Scots pine, representing different geographical origins of trees, exhibit distinct root endophytic fungal and bacterial communities and co-occurrence networks. It is hypothesized that (H_1_) the structure of root endophytic fungal and bacterial communities in *P. sylvestris* will vary according to tree ecotypes, influenced by variations in root biochemical phenotypes, such as starch, total non-structural carbohydrates, nitrogen, and carbon content, which are shaped by diverse climatic and geographical regions. Additionally, it is proposed (H_2_) that the association of root fungal and bacterial endophytes in *P. sylvestris* of different geographical origins is season-dependent, as the seasonal shifts in plant metabolic activity, likely induced the changes in root biochemistry. Furthermore, it is hypothesized (H_3_) that the co-occurrence of endophytic fungi and bacteria is influenced by the ecotypic/geographical origin of *P. sylvestris*, as their interaction may facilitate plant survival across various geographical locations under different environmental conditions.

## MATERIALS AND METHODS

### Study site, experimental design and sampling

**Fig 1:**
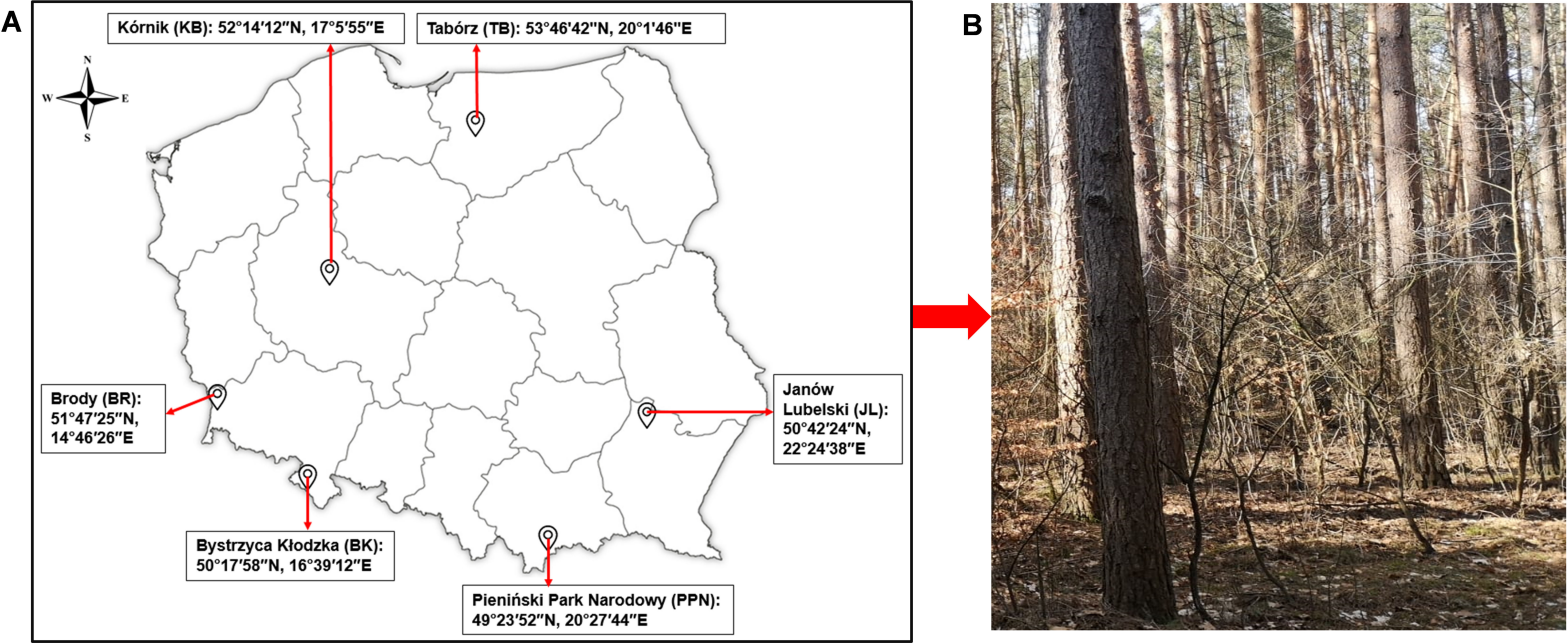
Illustration of *Pinus sylvestris* seeds from various geographical locations (**A**) and their cultivation in the common garden within the "Zwierzyniec" experimental forest in Kórnik (**B**).

The experimental common garden, hosting *P. sylvestris* trees of varied geographical origins in Poland (Fig. 1B), was established in the spring of 1967 within the "Zwierzyniec" experimental forest, which is affiliated with the Institute of Dendrology at the Polish Academy of Sciences in Kórnik, Poland. The seeds of *P. sylvestris* collected in 1965 from diverse geographical locations in Poland, including Kórnik (KB), Tabórz (TB), Brody (BR), Janów Lubelski (JL), Bystrzyca Kłodzka (BK), and Pieniński Park Narodowy (PPN) (Fig. 1A), were used for this purpose. The selection of seed origins was validated through consultations with local authorities at each collection site. The wide-ranging climatic conditions, in terms of mean annual precipitation and temperature (Table S1), of these locations have fostered the development of distinct *P. sylvestris* ecotypes for the common garden experiment. For this study, root samples were collected from a minimum of 10 individual trees across four blocks during the spring (19–22 April) and autumn (4–6 October) of 2021. Specifically, the samples were taken from approximately 30 lateral branches, each exceeding 2 mm in diameter, resulting in 120 root samples. These samples were immediately placed in an icebox and transported to the laboratory, where they were cleansed of soil particles in preparation for biochemical and microbial analyses. Microbial analysis subsamples were preserved at −20°C until further analysis.

### Climatic variables of the seed origin location of *P. sylvestris*

Climate data for the seed origin sites, spanning from 1956 to 1970, were obtained from the CHELSA database (49), renowned for its accurate precipitation predictions (49). This data set included historical records of average annual temperature and precipitation, alongside the highest and lowest temperatures during the warmest and coldest months, respectively. Additionally, the average temperatures for the wettest and driest quarters were recorded, as well as precipitation levels during the wettest and driest months and quarters. These comprehensive climate data, relevant to the seed origin sites, are detailed in Table S1.

### Root biochemistry analysis

The root subsamples underwent a drying process for 3 days at 65°C before biochemical analysis. The dried samples, including both fine and taproot fragments, were ground into powder using a Retsch MM 200 mill (Retsch, Haan, Germany). This powder was utilized for subsequent analyses. Carbon (C) and Nitrogen (N) concentrations were ascertained using an Elemental Combustion System CHNS-O 4010 (Costech Instruments, Italy/USA), calibrated with 2,5-(bis(5-tert-butyl-2-benzo-oxazol-2-yl) thiophene. Following the methodology described by Oleksyn et al. (50), non-structural carbohydrate concentrations (TNC — total soluble carbohydrates and starch) were estimated. Extraction involved a methanol–chloroform–water mixture (Avantor Performance Materials, Poland), with the anthrone reagent (Sigma) added to the extract, which was then heated in a water bath at 100°C for 12 min. The absorbance of the resulting mixture at 625 nm was measured within 30 min. The insoluble material was converted to glucose using amyloglucosidase (15 U/mL, Sigma, St Louis, MO, USA) and incubated for 24 h at 50°C. Glucose concentration was determined using the peroxidase–glucose oxidase-o-dianisidine dihydrochloride reagent (Sigma), with absorbance measured at 450 nm after 30 min of incubation at 25°C. Concentrations of soluble sugars and starch are expressed as a percentage of dry mass, using glucose as a standard. A detailed description of the root biochemical variables is provided in Table S2.

### Root processing and surface sterilization

For endophytic microbiome analysis, the roots were meticulously washed under running water to remove soil particles and litter debris. Cleaned roots, cut into 10-cm segments, underwent surface sterilization using two agents: 70% ethanol (C_2_H_5_OH) and 15% hydrogen peroxide (H_2_O_2_). The sterilization process involved agitating the roots in 15-mL flasks with 70% ethanol for 1 min, followed by 15% hydrogen peroxide for 2 min. After each sterilization step and again at the end, the roots were rinsed thrice with distilled water. The effectiveness of the surface sterilization was verified by incubating the water after the final rinse of roots at 25°C for up to 14 days on potato dextrose agar and nutrient agar media. The absence of microbial growth on these media confirmed the success of the sterilization. Sterilized root fragments were then stored at −20°C in Eppendorf tubes for subsequent DNA extraction.

### DNA extraction, PCR amplification, and sequencing

Genomic DNA was extracted from 0.3 g of frozen root using the GeneMatrix Plant and Fungi DNA purification kit (EUR_x_ Ltd, Gdansk, Poland). The roots were homogenized with liquid nitrogen in a sterilized mortar and pestle, then transferred to a tube for isolation, following the manufacturer’s protocol. DNA concentration and purity were assessed using a NanoDrop ND‐1000 Spectrophotometer (NanoDrop Technologies) and 1% agarose gel electrophoresis. Amplification and sequencing were conducted by Novogene Ltd (Cambridge Science Park, Cambridge, UK). The fungal internal transcribed spacer (ITS) and bacterial 16S rRNA gene were amplified using specific primer pairs: ITS1 and 1F for fungi, and 515F and 806R for bacteria, for Illumina MiSeq sequencing. PCR reactions utilized Phusion High-Fidelity PCR Master Mix (New England Biolabs) following the manufacturer’s instructions. PCR products were verified on 2% agarose gels, purified using the Qiagen Gel Extraction Kit (Qiagen, Germany), and libraries were prepared using the NEBNext Ultra DNA Library Prep Kit for Illumina. Library quality was assessed on the Qubit 2.0 Fluorometer (Thermo Scientific) and Agilent Bioanalyzer 2100 system, followed by sequencing on an Illumina platform, generating 250-bp paired-end reads.

### Bioinformatics analysis

Quality filtering of the raw tags was conducted using QIIME (V1.7.0) (51) under specific conditions to obtain high-quality clean tags (52). Quality control procedures were implemented to remove low-quality sequences, defined as those with an average quality score below 20, lacking a valid primer or barcode sequence, containing ambiguous bases, or shorter than 250 bp. The remaining sequences were compared with the reference databases, specifically the Unite database for fungi (https://unite.ut.ee/) (53) and the RDP database for bacteria (54), using the UCHIME algorithm (55) to identify and remove chimeric sequences (56). The non-chimeric sequences were clustered into operational taxonomic units (OTUs) at a 97% sequence similarity threshold using the UPARSE pipeline in USEARCH version 8.0 (57), following dereplication and the removal of singletons. Representative sequences for each fungal and bacterial OTU were aligned against the UNITE and RDP databases, respectively, using the Syntax algorithm in USEARCH version 8.0 (58). OTUs with fewer than 10 reads and those present in only one sample were excluded from the data sets to mitigate potential PCR or sequencing errors (59). To account for variations in sequence counts across samples, data were normalized to the size of the smallest sample using the subsample command in MOTHUR. All statistical analyses were conducted based on this normalized data set. Representative sequences for fungal and bacterial OTUs have been submitted to the European Nucleotide Archive (ENA) under accession numbers OY843772-OY844707 for fungi and OY971452-OY973596 for bacteria. Detailed information about endophytic fungi and bacteria OTUs in the present study is given in additional files as Tables S3 nd S4.

### Statistical analyses

Statistical analyses were performed in R version 3.3.2 (60). Tukey’s honestly significant difference (HSD) test was utilized to assess significant differences in root biochemical variables among different tree origin in each seasons. The Kruskal–Wallis test was applied to examine the effects of plant origin and season on the richness, Shannon, Simpson, and Pielou diversity indices of endophytic fungal and bacterial OTUs, as data transformation did not meet the assumptions of normality and homogeneity of variance. Conover’s test, facilitated by the posthoc.kruskal.conover.test function in the PMCMR package (61), was employed for multiple comparisons to identify significant differences in OTU richness among different *P. sylvestris* origins. The pheatmap function in the pheatmap package version 1.0.8 (62) was used to illustrate the relative abundances of the most abundant fungal endophytic OTUs across different *P. sylvestris* origins in both seasons (autumn and spring). Indicator species analysis for fungal and bacterial endophytic OTUs was conducted using the *indval* function in the labdsv package version 1.8–0 (63), with a focus on each plant origin in both seasons, considering OTUs with an *indval* value >0.6 and *P* < 0.05 as strong indicators. Dissimilarity matrices for endophytic fungal and bacterial communities were constructed using the Bray–Curtis method (64) after Hellinger transformation of the relative read numbers of OTUs. Permutational multivariate analysis of variance (PerMANOVA) was conducted using the adonis command in the vegan package based on 999 permutations (65) to evaluate the impact of plant origin and season on the community composition. Redundancy analysis, conducted using the *rda* function in the vegan package, visualized the variability in community composition across tree origins and seasons, incorporating Monte Carlo permutation tests with 999 permutations.

Co-occurrence networks, integrating fungal and bacterial communities for each tree origin in both seasons, were constructed following the methodology of Hartman et al. (39). Communities were normalized using the “trimmed mean of M-values” (TMM) method in the BioConductor package (66), and correlations between OTUs were calculated. Networks visualizing significant correlations (minimum threshold of *P* < 0.05, *r^2^* >0.9) were generated by Pearson’s correlation with the igraph package (67). Network topological properties, including node and edge numbers, average degree of co-occurrence, connectivity, and modularity, were analyzed using the igraph package (67–69).

## RESULTS

### Root biochemistry

The examination of root biochemical parameters revealed that the content of root starch and total non-structural carbohydrates (TNCs) was significantly higher in PPN compared with BK during the spring season. This significant difference was not observed in the autumn season. Furthermore, no significant differences in root starch and TNC content were found across the other tree ecotypes. In addition, analysis showed no significant differences in the total carbon (C) and nitrogen (N) contents in the roots among the various tree ecotypes, irrespective of the season (detailed results in Table S2).

### Characterization of Illumina sequencing data

From the initial 11,860,279 raw sequences derived from 120 samples, representing different tree origins across two seasons, 10,151,332 high-quality ITS sequences were obtained after quality filtering. These sequences were then clustered into 1,459 fungal OTUs at a 97% sequence similarity level. After excluding OTUs with fewer than 10 reads and those present only in one sample, the data set was refined to 936 fungal OTUs, comprising 10,110,616 reads. Upon normalization to the size of the smallest sample, a data set of 936 fungal OTUs with 4,428,120 reads was prepared for subsequent statistical analyses. Among these OTUs, 151 were found in ≥96 samples (frequency ≥82.0%), whereas the remaining 785 OTUs were detected in ≤95 samples (Fig. S1A). The identified fungal endophytes were predominantly from the Ascomycota (620 OTUs) and Basidiomycota (211 OTUs) phyla, with lesser contributions from Mortierellomycota (28 OTUs) and other phyla (Fig. S2A and S3A).

In the bacterial component, 9,125,636 high-quality 16S sequences were filtered from 11,825,930 raw sequences. These sequences were clustered into 3,798 bacterial OTUs at a 97% sequence similarity level. After removing OTUs with fewer than 10 reads and those present in only one sample, the data set was narrowed down to 2,168 bacterial OTUs, accounting for 3,332,587 reads. Following normalization based on the smallest sample size, a data set of 2,145 bacterial OTUs with 877,920 reads was prepared for further analysis. Of these, 319 OTUs were found in 69 or more samples (frequency ≥58.0%), with the remaining 1,826 OTUs detected in ≤68 samples (Fig. S1B). The bacterial endophytes were largely classified within the Proteobacteria (696 OTUs), Actinobacteriota (247 OTUs), and Bacteroidota (214 OTUs) phyla, among others (Fig. S2B and S3B).

### Diversity of endophytic fungi and bacteria

**Fig 2:**
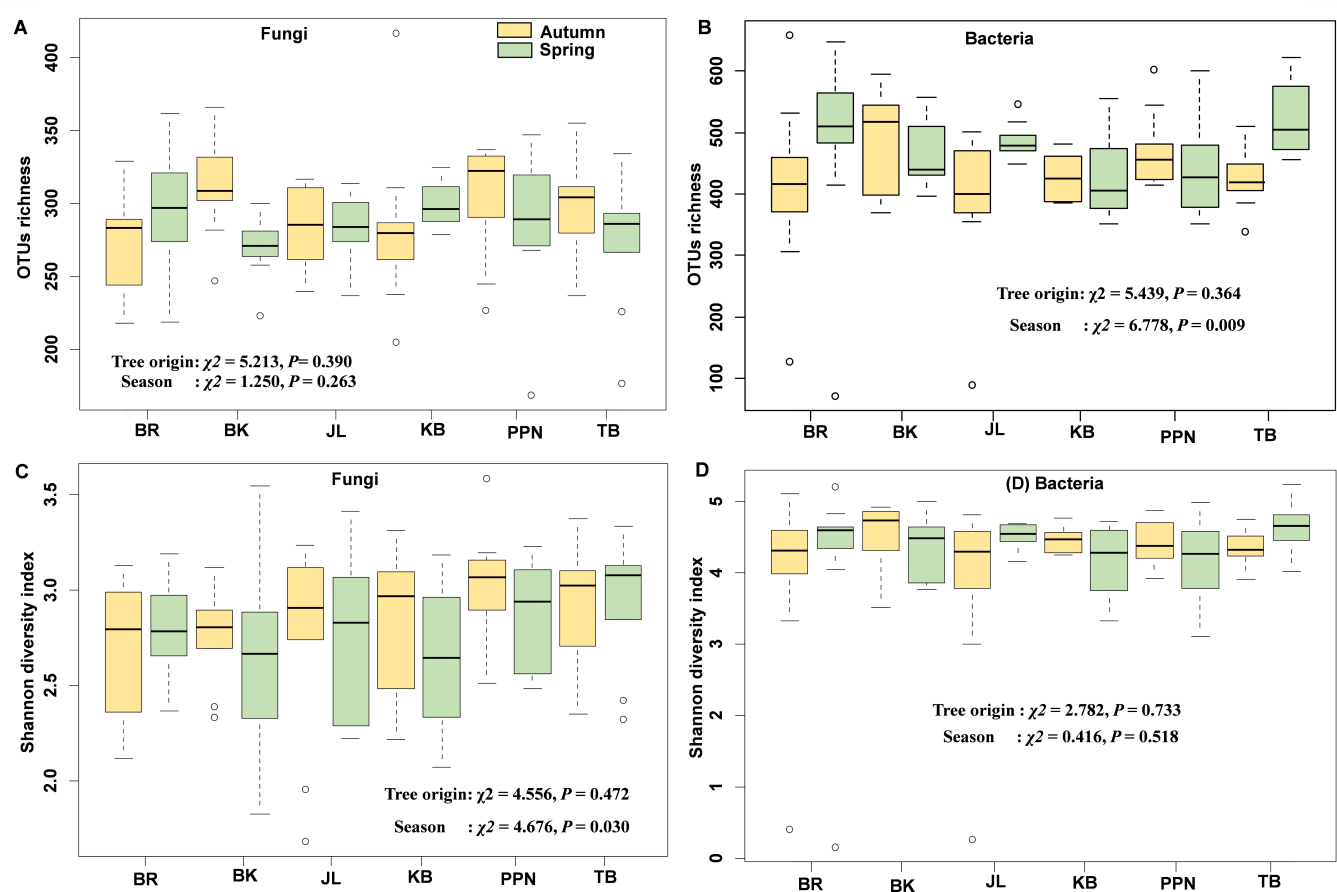
Operational taxonomic unit (OTU) richness (**A and B**) and Shannon diversity index (**C and D**) for fungal and bacterial endophytes across different *P. sylvestris* tree origins. The median value is depicted by the black line within each box plot. The Kruskal–Wallis test was utilized to examine the effects of tree origin and season on the OTU richness and Shannon diversity index of endophytic fungi and bacteria.

The diversity of endophytic fungi and bacteria in *P. sylvestris* was investigated using the Kruskal–Wallis test, which highlighted the distinct impacts of tree origin and season on microbial diversity. Tree origin did not significantly affect the OTU richness or diversity indices (Shannon, Simpson, and Pielou) for both fungal and bacterial endophytes. However, seasonal variations significantly influenced the richness of bacterial OTUs, the Shannon diversity index of fungal endophytes, and both the Simpson and Pielou diversity indices for fungal endophytes (Fig. 2A and B; Fig. S4A and B).

### Community composition of endophytic fungi and bacteria

**Fig 3:**
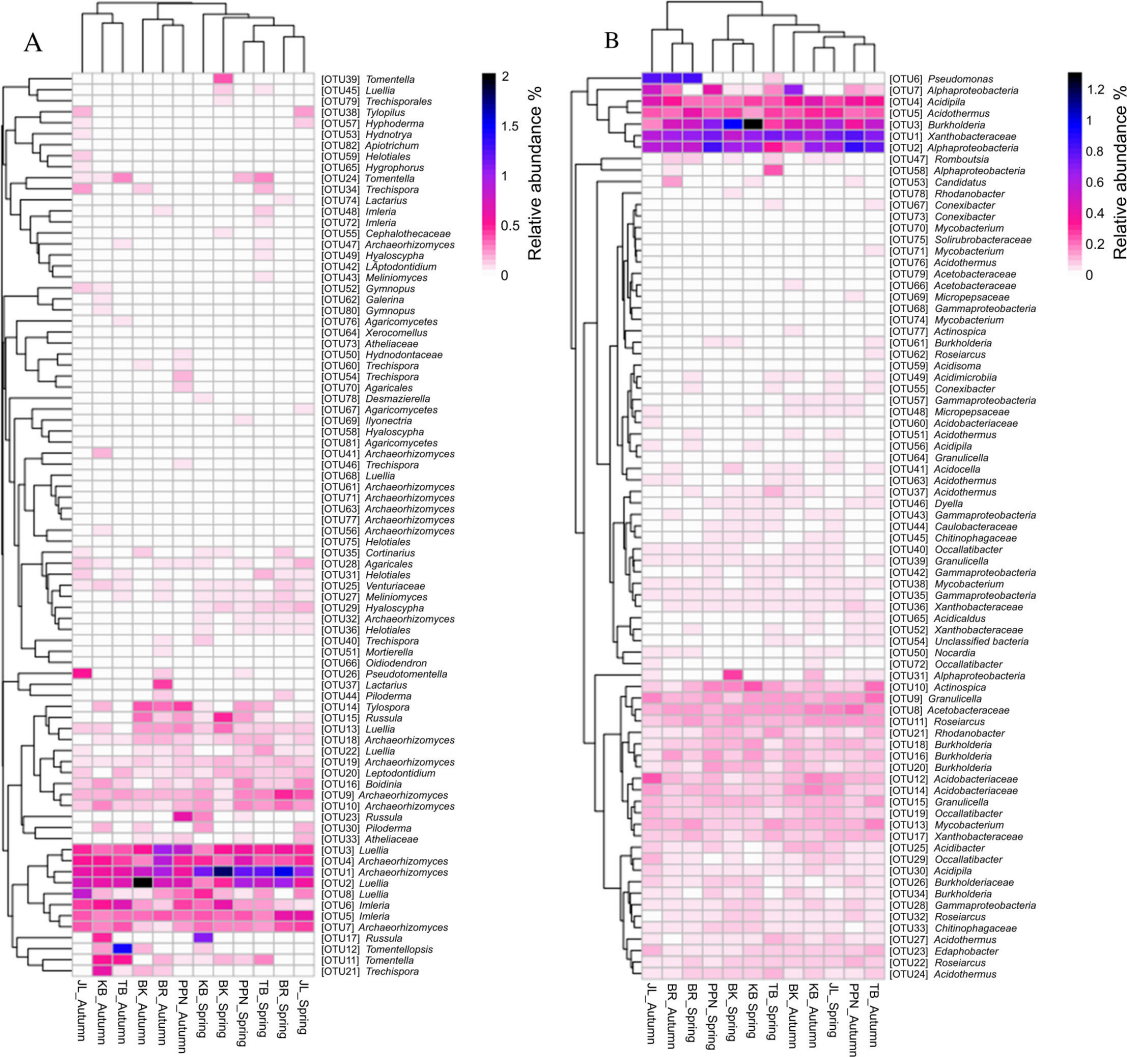
Heatmap representation of the distribution of the most abundant endophytic operational taxonomic units (OTUs, >4,000 reads for fungi; OTUs, >2,000 reads for bacteria) across different *P. sylvestris* tree origins in two seasons. (**A**) Fungal endophytes. (**B**) Bacterial endophytes. The heatmap cell color indicates the relative abundance of each endophytic OTU, with cluster analysis based on Bray–Curtis similarities.

The heatmap analysis demonstrated that the relative abundance of specific endophytic fungal and bacterial OTUs within *P. sylvestris* exhibited biases based on seasonal changes and geographical origins (Fig. 3A and B). During the spring season, the BK region was notably enriched with OTU39 (*Tomentella*) and OTU55 (*Cephalothecaceae*), whereas the autumn season saw a predominance of OTU50 (*Hydnodontaceae*), OTU54 (*Trechispora*), and OTU70 (*Agricales*) in PPN, alongside OTU51 (*Mortierella*) and OTU37 (*Lactarius*) in BR. JL presented a unique case by hosting OTU67 (*Agricomycetes*) exclusively in spring and retaining OTU38 (*Tylopilus*) and OTU57 (*Hydnotrya*) across both seasons, with OTU65 (*Hygrophorus*) and OTU59 (*Helotiales*) predominantly appearing in autumn. In the spring, KB was characterized by a high presence of OTU78 (*Desmazierella*), which shifted to OTU62 (*Galerina*) and OTU80 (*Gymnopus*) come autumn. This seasonal transition was mirrored in TB, which was marked by the significant autumnal presence of OTU76 (*Agricomycetes*) and OTU12 (*Tomentellopsis*). The bacterial OTU distribution followed a similar seasonal and geographical pattern. In BR and TB, OTU53 (*Candidatus*) and OTU71 (*Mycobacterium*), respectively, were predominantly observed in the autumn, whereas OTU58 (*Alphaproteobacteria*) showed abundance in spring, and OTU67 (*Conexibacter*) was present in both seasons within TB. The spring season in JL was marked by an abundance of OTU64 (*Granulicella*), transitioning to OTU60 (*Acidobacteraceae*) in autumn. BK displayed a notable abundance of OTU78 (*Rhodanobacter*) in spring and OTU66 (*Acidobacteriaceae*) in autumn. Exclusively in the autumn, PPN harbored OTU69 (*Micropepsaceae*), highlighting the unique microbial communities associated with different *P. sylvestris* origins across the changing seasons.

**Fig 4:**
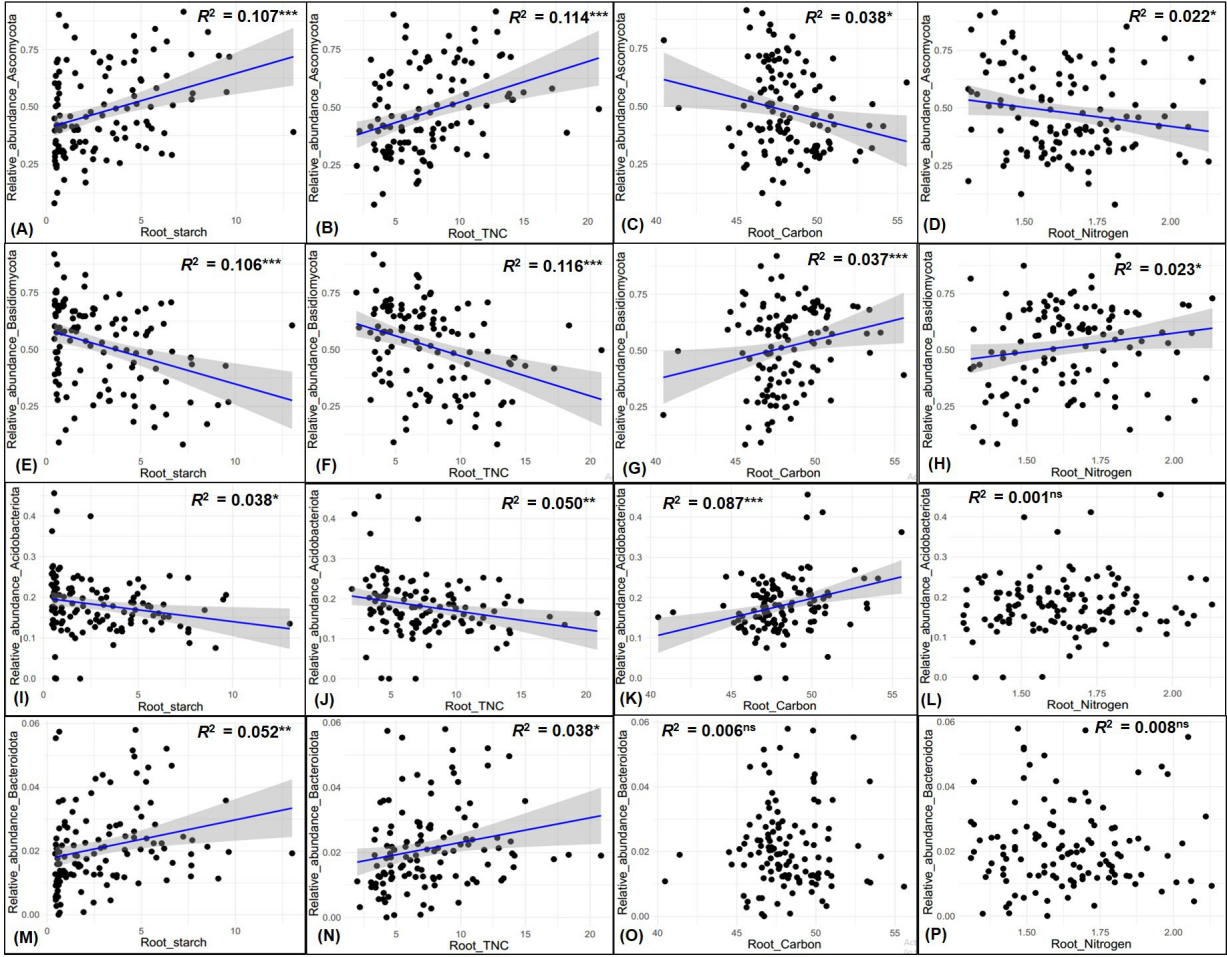
Linear relationships between root biochemical variables and endophytic fungal and bacterial phyla. Only phyla significantly correlated with at least one biochemical variable are shown. TNC represents total non-structural carbohydrates; Root C denotes root carbon; Root N signifies root nitrogen.

Indicator species analysis identified significant endophytic fungal and bacterial OTUs within *P. sylvestris*, revealing notably higher distributions in PPN during autumn for fungi and in TB during spring for bacteria, relative to other origins and seasons (Fig. S5A and B). Furthermore, strong fungal endophytic indicator OTUs, with *Indval* values exceeding 0.6 and belonging to the Basidiomycota phylum, such as OTU142 and OTU270, were predominantly found in PPN during the autumn season. In contrast, OTU39 and OTU79 were prevalent in PPN during the spring. OTU12 and OTU87 were distinctly present in JL and KB, respectively, during spring, whereas OTU26 was identified in TB during the autumn season. Conversely, strong indicators within the Ascomycota phylum, including OTU56, OTU69, and OTU110, were notably observed in BK during the spring season. OTU82 and OTU115 were identified in BR across both spring and autumn seasons, respectively (Table 1). Furthermore, a selection of strong bacterial endophytic indicators, namely OTU968, OTU687, OTU1039, OTU825, OTU672, and OTU518, associated with diverse phyla, such as Verrucomicrobiota, Actinobacteriota, Firmicutes, Bacteroidota, and Proteobacteria, were exclusively observed in TB during the spring season (Table 1). Simple linear regression analysis demonstrated a positive correlation between the relative abundance of Ascomycota and root starch and total non-structural carbohydrates (TNC), alongside a negative correlation with total root carbon (C) and nitrogen (N) (4A, B, C and D). Inversely, the relative abundance of Basidiomycota exhibited a negative correlation with root starch and TNC but showed a positive correlation with total root C and N (4E, F, G and H). Within the bacterial community, Acidobacteriota’s relative abundance was negatively correlated with root starch and TNC, yet positively correlated with root C (4I, J, and K). Furthermore, the relative abundance of Bacteroidota positively correlated with root starch and TNC (Fig. 4M and N).

**TABLE 1:** Strong endophytic fungal and bacterial indicator operational taxonomic units (OTUs) in *P. sylvestris*

Name	*P*-value	*Indval* value (> 0.6)	Tree origin	Season	Taxonomic position (Phylum)	Taxonomic position (Genus)
Fungi						
OTU39	0.001	0.769	PPN	Spring	Basidiomycota	*Tomentella*
OTU142	0.001	0.740	PPN	Autumn	Basidiomycota	*Cortinarius*
OTU79	0.001	0.677	PPN	Spring	Basidiomycota	Unknown
OTU270	0.001	0.671	PPN	Autumn	Basidiomycota	*Coniophora*
OTU56	0.001	0.873	BK	Spring	Ascomycota	*Archaeorhizomyces*
OTU69	0.001	0.810	BK	Spring	Ascomycota	*Ilyonectria*
OTU110	0.003	0.643	BK	Spring	Ascomycota	Unknown
OTU82	0.001	0.674	BR	Spring	Ascomycota	*Apiotrichum*
OTU115	0.037	0.637	BR	Autumn	Ascomycota	*Archaeorhizomyces*
OTU12	0.001	0.689	JL	Spring	Basidiomycota	*Tomentellopsis*
OTU26	0.001	0.666	TB	Autumn	Basidiomycota	*Pseudotomentella*
OTU87	0.031	0.643	KB	Spring	Basidiomycota	*Peniophorella*
Bacteria						
OTU968	0.001	0.700	TB	Spring	Verrucomicrobiota	Unknown
OTU498	0.001	0.652	TB	Spring	Unidentified	Unknown
OTU687	0.001	0.603	TB	Spring	Actinobacteriota	*Pseudonocardia*
OTU1039	0.001	0.600	TB	Spring	Firmicutes	*Blautia*
OTU825	0.001	0.600	TB	Spring	Bacteroidota	Unknown
OTU672	0.001	0.605	TB	Spring	Proteobacteria	Unknown
OTU518	0.001	0.601	TB	Spring	Firmicutes	*Parvimonas*

**Fig 5:**
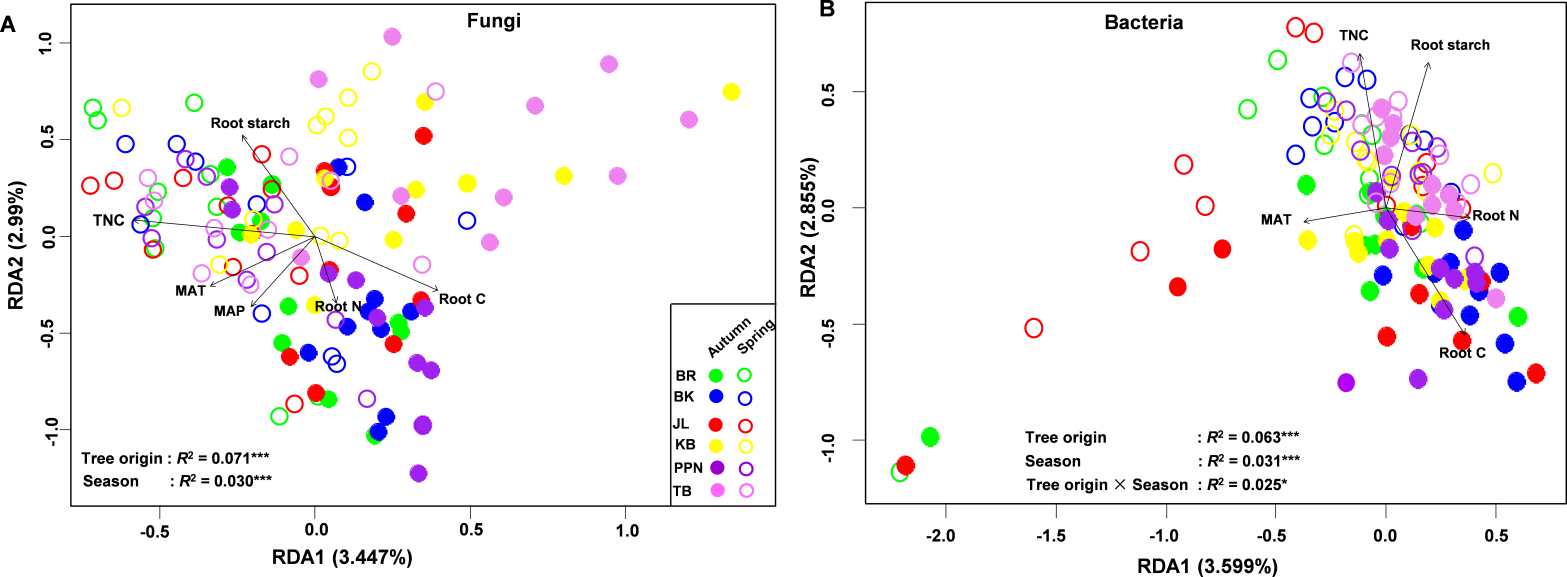
Redundancy analysis (RDA) illustrating the bioplot variability of fungal (**A**) and bacterial (**B**) endophytic community compositions in *P. sylvestris* across different tree origins and seasons. Permutational analysis of variance (PerMANOVA) and RDA assess the influence of tree origin and season on endophytic fungal and bacterial community compositions relative to significant root and climatic variables (*P < 0.05, **P < 0.01, ***P < 0.001). TNC, total non-structural carbohydrates; Root C, root carbon; Root N, root nitrogen; MAT, mean annual temperature; MAP, mean annual precipitation of the tree origin’s geographical area.

Redundancy analysis (RDA) and permutational multivariate analysis of variance (PerMANOVA) highlighted the significant influence of seasonal variations and tree origin on the composition of fungal endophytic communities within *P. sylvestris* (Fig. 5A). Specifically, the season had a notable impact (*F* = 3.846, *R*^2^ = 0.030, *P* = 0.001), as did the tree origin (*F* = 1.822, *R*^2^ = 0.071, *P* = 0.001). Additionally, the analyses revealed significant correlations between the fungal endophytic community composition and root biochemical traits, including starch, total non-structural carbohydrates (TNC), carbon (C), and nitrogen (N), as well as climatic variables from the tree’s origin, such as mean annual precipitation and temperature (Fig. 5A; Table 2). The bacterial endophytic community composition was also significantly affected by seasonal changes (*F* = 4.038, *R*^2^ = 0.031, *P* = 0.001) and the tree’s origin (*F* = 1.626, *R*^2^ = 0.063, *P* = 0.001), with a notable interaction between these two factors (*F* = 1.398, *R*^2^ = 0.054, *P* = 0.011). Similar to the fungal community, the composition of the bacterial endophytic community was significantly correlated with root starch, TNC, nitrogen (N), carbon (C), and the mean annual temperature associated with the tree’s geographical origin (Fig. 5B; Table 2).

**TABLE 2:** The relative impact of root biochemical and plant origin climatic variables on the composition of endophytic fungal and bacterial communities in *P. sylvestris*, as revealed by permutational multivariate analysis of variance (PerMANOVA)^*a*^

Variables		Fungi			Bacteria		
	df	*F*	*R^2^*	*p*	*F*	*R^2^*	*p*
TNC	1	3.576	0.029	0.001	1.973	0.016	0.023
Root C	1	2.314	0.019	0.001	1.933	0.016	0.038
Root N	1	1.555	0.013	0.034	1.523	0.011	0.046
Root starch	1	3.293	0.027	0.001	1.970	0.016	0.025
MAT	1	1.662	0.013	0.018	2.078	0.017	0.015
MAP	1	1.633	0.013	0.030	1.109	0.009	0.267

^*a*^ TNC, total non-structural carbohydrates; Root C, root carbon; Root N, root nitrogen; MAT, mean annual temperature of tree origin geographical area; MAP, mean annual precipitation of tree origin geographical area.

### Co-occurrence network of fungal and bacterial community

**Fig 6:**
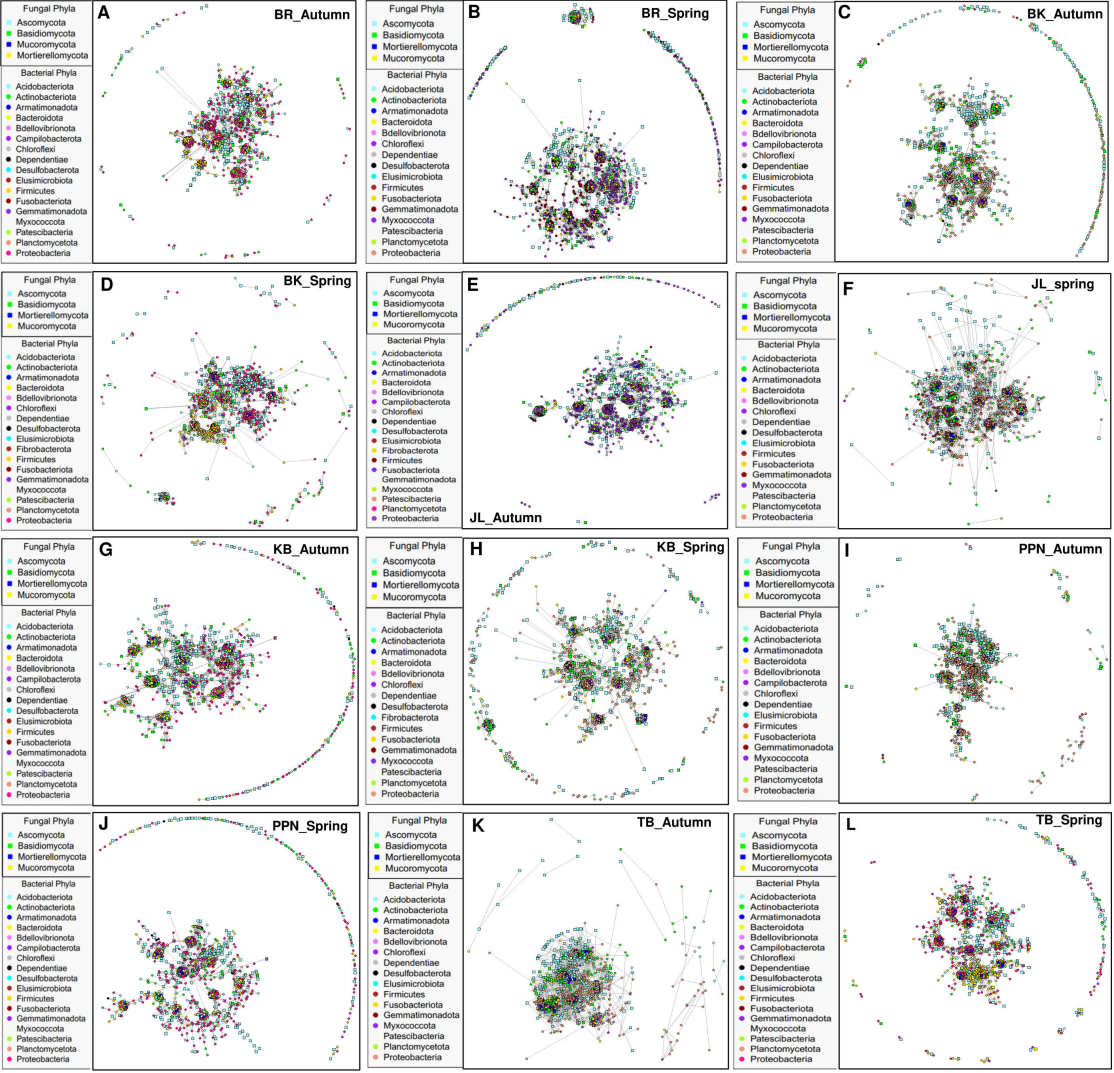
Co-occurrence networks illustrating positive correlations between endophytic fungal and bacterial OTUs in *P. sylvestris* originating from different geographical regions across two seasons. Bacterial and fungal nodes (OTUs) are represented by points and squares, respectively, with lines (edges) indicating significant correlations between nodes.

**Fig 7:**
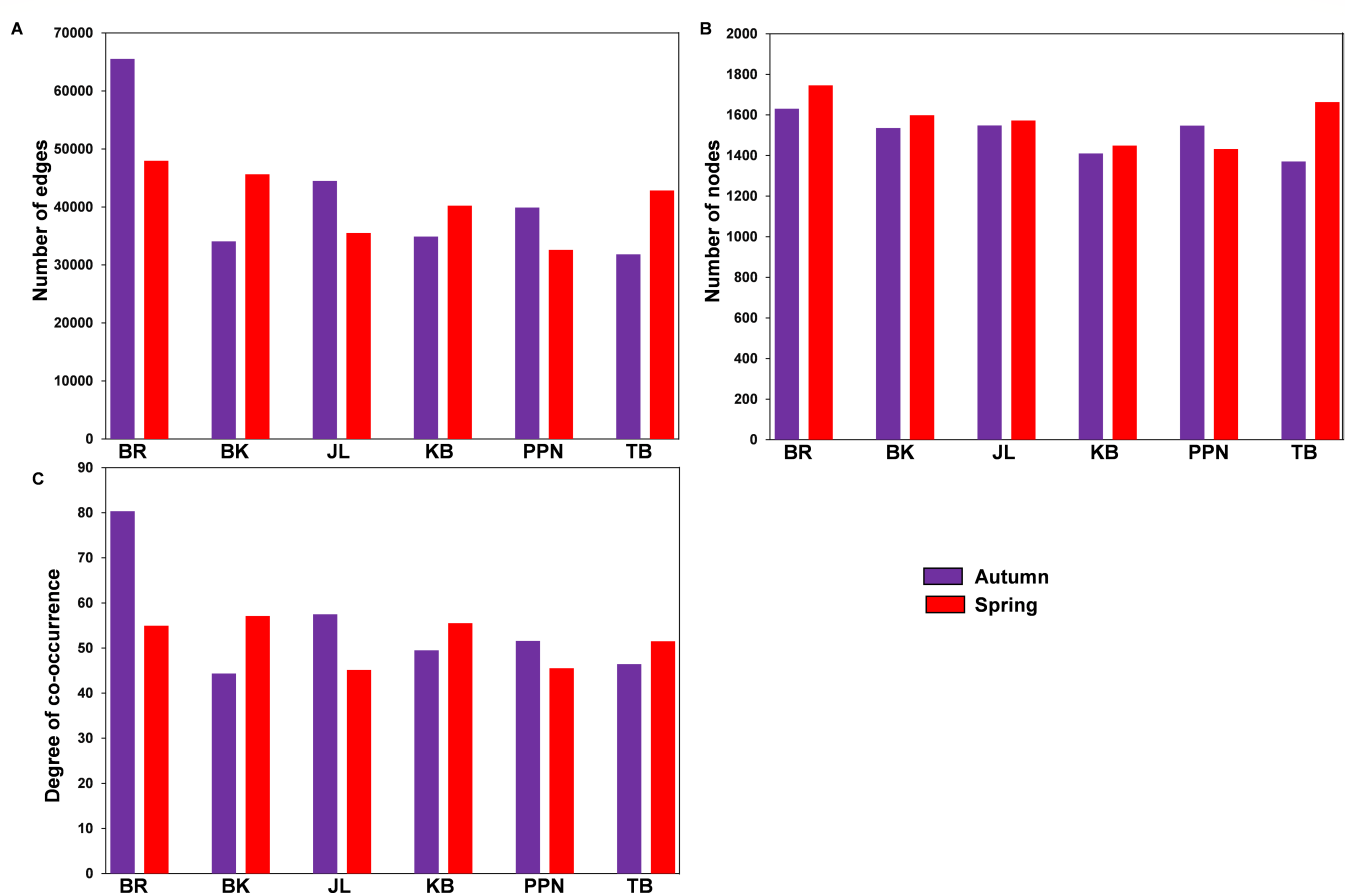
Topological properties of co-occurrence networks for fungal and bacterial endophytes in *P. sylvestris* originating from various geographical regions across two seasons. The complexities of these networks are highlighted by the number of edges (**A**), nodes (**B**), and the degree of co-occurrence (**C**).

**Fig 8:**
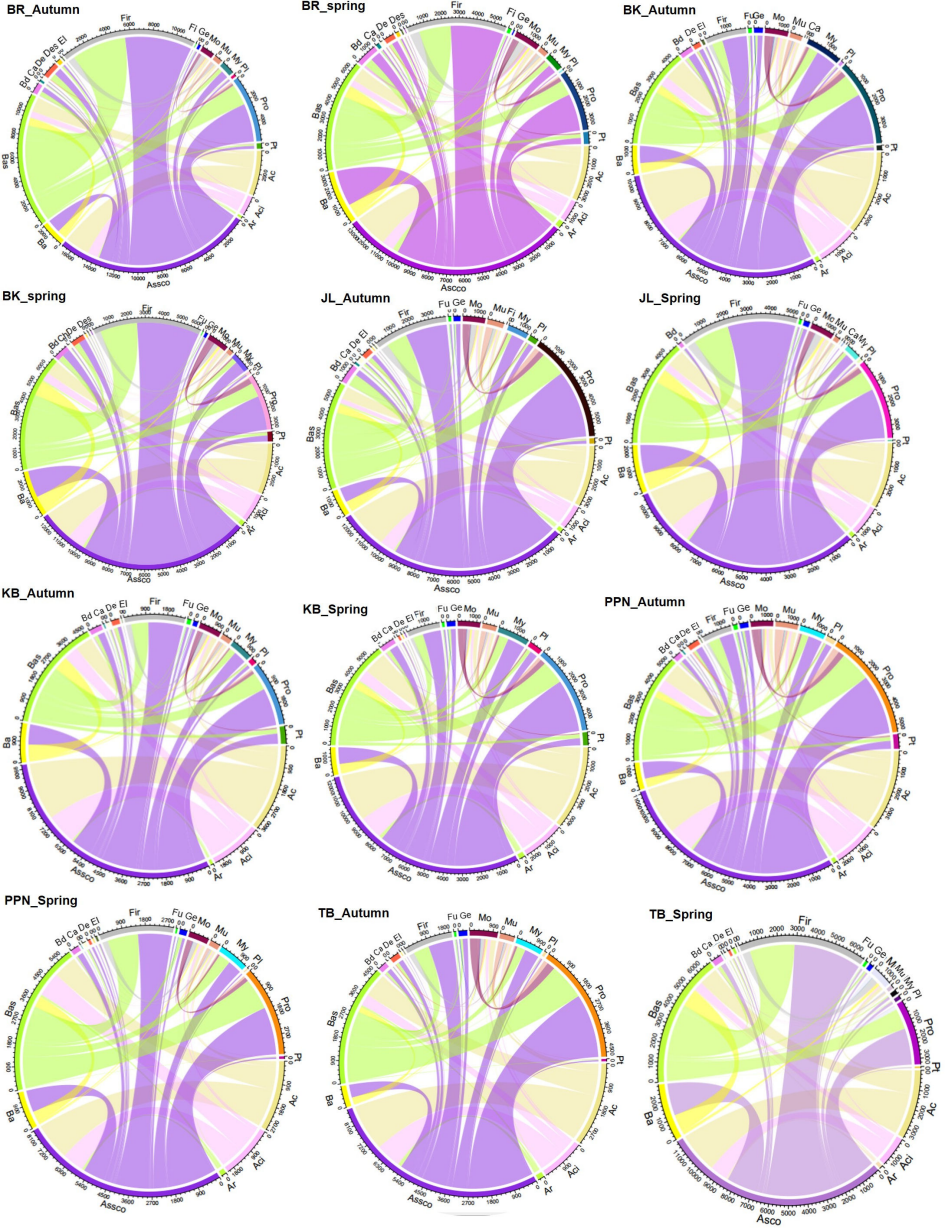
Interrelationships between fungal and bacterial phyla based on the co-occurrence network matrix across different tree origins in two seasons. The circle’s numbers denote the co-occurrence frequency for each phylum, with the chord thickness representing the co-occurrence strength between specific fungal and bacterial phyla pairs. Assco, Ascomycota; Ar, Armatimonadota; Ac, Actinobacteriota; Aci, Acidobacteriota; Bas, Basidiomycota; Ba, Bacteroidota; Bd, Bdellovibrionota; Ca, Campilobacterota; Ch, Chloroflexi; De, Dependentiae; Des, Desulfobacterota; El, Elusimicrobiota; Fir, Firmicutes; Fi, Fibrobacterota; Fu, Fusobacteriota; Ge, Gemmatimonadota; Mo, Montrilomycota; Mu, Mucoromycota; My, Myxococcota; Pro, Proteobacteria; Pl, Planctomycetota; Pt, Patescibacteria.

Significant positive correlations were identified between endophytic OTUs of fungi and bacteria across various geographical origins of *P. sylvestris*, spanning two distinct seasons. This analysis revealed variations in network complexity, characterized by the number of edges, nodes, average degree of co-occurrence, connectivity, and modularity (Fig. 6 and 7; Table S5). The co-occurrence networks of fungal and bacterial endophytes displayed considerable diversity across geographical origins and seasons. Notably, the BR region demonstrated the most intricate interaction patterns, especially in the autumn season, with the highest count of edges and an elevated average degree of co-occurrence compared with other regions. This increased complexity suggests a densely interconnected web of interactions between fungal and bacterial communities in the endophytic ecosystem of BR (Fig. 6 and 7A and C). Moreover, BR consistently exhibited the greatest number of nodes throughout both seasons, indicative of a broad spectrum of endophytic OTUs (Fig. 6 and 7B). Other regions displayed unique network characteristics; for example, BK and JL were distinguished by the highest modularity during autumn and spring, respectively (Table S5). These findings underscore the profound impact of geographical origin on the dynamics of symbiotic relationships between endophytic fungi and bacteria in *P. sylvestris*, with the BR region showcasing particularly complex network structures, especially during the autumn season. Additionally, the analysis of inter-kingdom co-occurrence revealed variations in the interactions among fungal and bacterial phyla across different pine origins and seasons (Fig. 8). For instance, the frequency and strength of co-occurrence between Ascomycota and Basidiomycota with Firmicutes were notably higher in the BR region during both spring and autumn, as well as in BK, JL, and TB during spring, compared with PPN and KB in both seasons, and BK and TB in autumn (Fig. 8). This variation in microbial interactions across regions and seasons reflects the complex ecological networks within the endophytic communities of *P. sylvestris*, influenced by the specific environmental and climatic conditions of each geographical origin.

## DISCUSSION

### *P. sylvestris* tree origin (ecotype) influenced root endophytic community

The study’s findings corroborate the initial hypothesis (H_1_) that the community composition of endophytic fungi and bacteria in *P. sylvestris* is influenced by seed origin. This aligns with previous research indicating that genotypic variations within tree species affect the composition of root fungal endophytic and ectomycorrhizal communities (21, 24, 67, 70, 71). The observed dissimilarities in community composition among different ecotypes of *P. sylvestris* are likely attributed to variations in the relative abundance of endophytic fungi and bacteria. These variations are potentially due to the differences in host root biochemical and nutritional status resulting from the host’s evolutionary adaptations to diverse climatic conditions (19, 20). Such intraspecific variability in functional traits, related to water and nutrient uptake and physiological responses to abiotic stress, facilitates a species' survival across wide geographic ranges by adapting to various climates (72–76). Laboratory studies further support the notion that intraspecific variation can influence root endophyte communities (77). In this investigation, the community composition of root endophytic fungi and bacteria was found to correlate with host root biochemical phenotypes, such as total non-structural carbohydrate (TNC), starch, nitrogen (N), phosphorus (P), carbon (C), and climatic variables from the host origin, including mean annual precipitation (MAP) and temperature (MAT). This highlights the significant impact of diverse climatic conditions linked to seed origins on the microbiome of mature *P. sylvestris* trees. There is considerable variability in MAP and MAT associated with the seeds' geographical origins (Table S1), which significantly correlates with the community composition of fungi and bacteria in our study. Variations in root biochemical parameters, as highlighted in previous studies, play a pivotal role in shaping the microbial community, with organic carbon and total nitrogen content being key factors in microbial recruitment (20). The study revealed that Basidiomycota OTUs, indicator OTUs of high starch and TNC conditions, were predominantly observed in trees from PPN origin. In contrast, Ascomycota OTUs, indicator OTUs of low starch and TNC conditions, were associated with trees from BK origin. This pattern reflects the differential responses of Basidiomycota and Ascomycota to plant-generated carbon metabolites, likely indicative of their symbiotic or saprotrophic nature. For instance, Basidiomycota OTU39 (*Tomentella*) and OTU79 (*Trechisporales*) showed lower abundance in PPN, whereas Ascomycota OTU56 (*Archaeorhizomyces*) and OTU69 (*Ilyonectria*) decreased in BK. This suggests that the root organic carbon metabolites from different tree origins influence these fungal groups, establishing them as indicator or rare species within their respective host environments. Furthermore, the study observed that trees originating from regions with higher precipitation and cooler temperatures (e.g., PPN) tend to store more photosynthetic carbon (e.g., starch or TNC) (Table S2). This storage potentially reduces colonization by symbiotic fungi (e.g., ectomycorrhiza) in favor of other plant growth-promoting endophytic microorganisms. Additionally, the positive and negative correlations of bacterial phyla Acidobacteriota and Bacteroidota, respectively, with root starch and TNC, indicate differential responses of these bacterial groups to plant-produced carbohydrates. Contrary to the initial hypothesis, OTU richness of endophytic fungi and bacteria was not significantly affected by the tree origin or ecotype. This suggests that the species pools of inherent microbiomes within an intra-species tree may be more homogeneous in a common garden setting than in natural habitats (78). However, bacterial OTU richness and fungal diversity indices (Shannon, Simpson, and Pielou) were influenced by season, indicating the stability of species diversity pools across different *P. sylvestris* ecotypes. Although tree origin affects the relative abundance of certain fungi and bacteria across seasons, it does not lead to significant species turnover or decline. This resilience in the microbial communities suggests an adaptation mechanism that maintains species presence despite fluctuations in abundance.

### Variation of root endophytic abundance in different ecotype *P. sylvestris* depended on growing season

Consistent with the second hypothesis (H_2_), this study observed that the relative abundance of endophytic fungi and bacteria in various ecotypes of *P. sylvestris* varied with the growing season, reflecting the seasonal shifts in endophytic community composition attributable to differences in plant metabolic activity (29). Specifically, the study identified a seasonal bias in the relative abundance of certain endophytic fungal (*Tomentella*, *Cephalothecaceae*, *Hydnodontaceae*, *Agricales*, *Trechispora*, *Russula*, etc.) and bacterial (*Alphaproteobacteria*, *Conexibacter*, *Mycobacterium*, *Granulicella*, *Acidobacteraceae*, *Rhodanobacter*, *Actinospica*, and *Micropepsaceae*) OTUs across different origins of pine in varying seasons. The fluctuating abundance of these endophytic microorganisms could be attributed to a combination of host genetic characteristics unique to different origins (79–81), and environmental influences, such as the available pool of colonizers, site conditions, and climatic factors, such as temperature and water availability (82–84). These factors collectively impact the establishment of microorganisms on host roots across different seasons, in line with the substantial variability in precipitation and temperature observed between wetter and drier, as well as warmer and colder periods, among the various ecotypes of *P. sylvestris* (Table S1). Indicator species analysis further supported the notion that the associations of endophytic fungi and bacteria with different ecotypes/origins of *P. sylvestris* are season-dependent. For instance, a higher number of indicator OTUs of fungal endophytes was observed in *P. sylvestris* originating from PPN in autumn, followed by BK, BR, JL, and KB in spring, and TB in autumn. Conversely, a higher number of significant bacterial endophytic indicator OTUs were distributed in TB in spring, followed by BK in autumn, and then JL, KB, PPN, and BR in spring. These significant indicator species, either rare or lower in abundance in their specific origins during different seasons, may result from adaptive divergence in *P. sylvestris* due to molecular variation (85), which influences root biochemistry and microbial abundance. The correlation between the relative abundance of certain fungal (e.g., Ascomycota and Basidiomycota) and bacterial phyla (e.g., Acidobacteria and Bacteroidota) with root starch and TNC supports this assumption. Specifically, pine plants from cooler and wetter regions, such as PPN exhibited higher root starch and TNC levels in spring than in autumn (Table S2). Moreover, the variation in endophytic microbes marginally depended on root C and N concentrations, as certain fungal and bacterial phyla showed positive or negative correlations with these parameters. This might be related to the varying concentrations of C and N in different tree origins across seasons. For example, pines from the cooler and wetter regions of PPN contained relatively higher concentrations of root N and C in autumn and lower levels in spring (Table S2). The presence of a higher number of indicator or rare OTUs (e.g., Basidiomycota OTUs) in pines from the Pieniny National Park (PPN) during autumn suggests that some symbiotic Basidiomycota OTUs (e.g., *Tomentella*, *Trechisporales*) may promote plant growth by supplying more N in exchange for C, as this phylum is positively correlated with root C and N. Conversely, the abundance of indicator OTUs of bacteria in pines from Tabórz (TB) in spring might be due to TB producing more C resources in spring than in autumn, as some bacterial phyla (e.g., Acidobacteria and Bacteroidota) were positively or negatively correlated with root starch, TNC, or root C content.

### Co-occurrence of endophytic fungi and bacteria across ecotype of *P. sylvestris* and season

The tree origin of *P. sylvestris* had a pronounced impact on the co-occurrence networks of endophytic fungi and bacteria, particularly in terms of average edge number, node number, average degree of co-occurrence, connectivity, and modularity. This finding aligns with our third hypothesis (H_3_) and indicates a robust interplay between fungal and bacterial OTUs within the *P. sylvestris* from the BR region. This region exhibited enhanced network complexity, characterized by increased edges and connectivity, suggesting a more intricate coupling between fungal and bacterial OTUs (43). The presence of a higher number of positively associated bacteria and fungi in the BR network points to increased cooperative interactions and exchange events (86). A modularity index above 0.4, indicative of a modular network configuration (87), was observed across all networks, with particularly high modularity noted in the BK region during autumn and the JL region in spring. Previous observations by Krause et al. (88) suggest that higher modularity, denoted by numerous clusters of interacting species, may bolster the resilience of microbial communities against environmental changes and contribute to the stability of their interaction networks. The variations in network metrics among different *P. sylvestris* origin populations could stem from ecological processes at the microbial level, influenced by plant presence and evolutionary adaptations to varying environmental conditions (89). The pronounced degree of co-occurrence in the BR region underscores the significance of inter-kingdom interactions among fungal and bacterial communities in this particular geographical locale of *P. sylvestris*. Moreover, the study observed diverse inter-phyla co-occurrence patterns across different origins and seasons. For instance, the frequency and strength of co-occurrence between Ascomycota and Basidiomycota with Firmicutes were notably higher in the BR region during both spring and autumn compared with other origins. This pattern could be attributed to variations in photosynthetically derived carbon, such as root starch and TNC, which were higher in cooler and wetter regions (e.g., PPN) than in others (Table S2), potentially influencing the dynamics of endophyte cooperation for nutrient acquisition. The indicator species analysis highlighted specific fungal and bacterial OTUs as strong indicators within certain origins and seasons, such as a higher number of fungal indicators in PPN during autumn and bacterial indicators in TB during spring. These findings suggest that adaptive divergence in *P. sylvestris*, possibly due to molecular variations influencing root biochemistry, significantly affects microbial abundance. For example, the relative abundance of certain fungal (e.g., Ascomycota and Basidiomycota) and bacterial phyla (e.g., Acidobacteria and Bacteroidota) showed significant correlations with root starch and TNC. Additionally, variations in endophytic microbes appeared to be marginally influenced by root carbon (C) and nitrogen (N) concentrations, with some fungal and bacterial phyla displaying positive or negative correlations with these root biochemical parameters.

### Conclusions

This investigation provides the first insights into how the geographical origin of *P. sylvestris* seeds affects the root endophytic fungal community and their co-occurrence networks across different seasons. The study established that the composition of endophytic fungi and bacteria in *P. sylvestris* trees is shaped by both the tree’s origin and the season, and is closely linked to root biochemical traits, such as starch, total non-structural carbohydrates, carbon, and nitrogen. The observed variability in the relative abundance of key fungal and bacterial taxa across different seasons underscores the influence of tree origin on microbial community dynamics. Furthermore, the co-occurrence networks of fungal and bacterial endophytes within *P. sylvestris* exhibited variations in network complexity across geographical origins and seasons. The ecotypic differences in host root biochemical phenotypes, driven by evolutionary adaptation to geographical and climatic conditions, play a pivotal role in shaping the root endophytic community assembly of *P. sylvestris*. Future research in more natural settings, exploring how different *P. sylvestris* ecotypes select root endophytes through soil communities, will be crucial for validating the current findings on root endophytic community assembly.

## SUPPLEMENTAL MATERIAL

Supplemental material spectrum.00807-24-s0001.docx Fig. S1 to S5; Tables S1 and S2, S5.

Table S3 spectrum.00807-24-s0002.xlsx Molecular identification of endophytic fungi at a 97% sequence identity level in this study.

Table S4 spectrum.00807-24-s0003.xlsx Molecular identification of endophytic bacteria at a 97% sequence identity level in this study.

ASM does not own the copyrights to Supplemental Material that may be linked to, or accessed through, an article. The authors have granted ASM a non-exclusive, world-wide license to publish the Supplemental Material files. Please contact the corresponding author directly for reuse.

## References

[1] Dastogeer KMG, Tumpa FH, Sultana A, Akter MA, Chakraborty A. 2020. Plant microbiome–an account of the factors that shape community composition and diversity. Curr Plant Biol 23:100161. doi:10.1016/j.cpb.2020.100161

[2] Tedersoo L, Bahram M, Zobel M. 2020. How mycorrhizal associations drive plant population and community biology. Science 367:eaba1223. doi:10.1126/science.aba1223 32079744

[3] Wężowicz K, Rozpądek P, Turnau K. 2017. Interactions of arbuscular mycorrhizal and endophytic fungi improve seedling survival and growth in post-mining waste. Mycorrhiza 27:499–511. doi:10.1007/s00572-017-0768-x 28317065 PMC5486607

[4] Pal G, Saxena S, Kumar K, Verma A, Sahu PK, Pandey A, White JF, Verma SK. 2022. Endophytic Burkholderia: multifunctional roles in plant growth promotion and stress tolerance. Microbiol Res 265:127201. doi:10.1016/j.micres.2022.127201 36167006

[5] Hosseyni Moghaddam MS, Safaie N, Soltani J, Hagh-Doust N. 2021. Desert-adapted fungal endophytes induce salinity and drought stress resistance in model crops. Plant Physiol Biochem 160:225–238. doi:10.1016/j.plaphy.2021.01.022 33517220

[6] Kamran M, Imran QM, Ahmed MB, Falak N, Khatoon A, Yun BW. 2022. Endophyte-mediated stress tolerance in plants: a sustainable strategy to enhance resilience and assist crop improvement. Cells 11:3292. doi:10.3390/cells11203292 36291157 PMC9600683

[7] Geisen S, Hooven FC, Kostenko O, Snoek LB, Putten WH. 2020. Fungal root-endophytes influence plants in a species-specific manner that depends on plant's growth stage. Dryad Dig Rep. doi:10.5061/dryad.jq2bvq87v

[8] Vahter T, Bueno CG, Davison J, Herodes K, Hiiesalu I, Kasari‐Toussaint L, Oja J, Olsson PA, Sepp S, Zobel M, Vasar M, Öpik M. 2020. Co-introduction of native mycorrhizal fungi and plant seeds accelerates restoration of post-mining landscapes. J Appl Ecol 57:1741–1751. doi:10.1111/1365-2664.13663

[9] McGuire KL. 2007. Common ectomycorrhizal networks may maintain monodominance in a tropical rain forest. Ecology 88:567–574. doi:10.1890/05-1173 17503583

[10] Werner GDA, Zhou Y, Pieterse CMJ, Kiers ET. 2018. Tracking plant preference for higher-quality mycorrhizal symbionts under varying CO_2_ conditions over multiple generations. Ecol Evol 8:78–87. doi:10.1002/ece3.3635 29321853 PMC5756855

[11] Wang Y-L, Gao C, Chen L, Ji N-N, Wu B-W, Li X-C, Lü P-P, Zheng Y, Guo L-D. 2019. Host plant phylogeny and geographic distance strongly structure Betulaceae-associated ectomycorrhizal fungal communities in Chinese secondary forest ecosystems. FEMS Microbiol Ecol 95:fiz037. doi:10.1093/femsec/fiz037 30889238

[12] Wang B, Sugiyama S. 2020. Phylogenetic signal of host plants in the bacterial and fungal root microbiomes of cultivated angiosperms. Plant J 104:522–531. doi:10.1111/tpj.14943 32744366

[13] Aavik T, Träger S, Zobel M, Honnay O, Van Geel M, Bueno CG, Koorem K. 2021. The joint effect of host plant genetic diversity and arbuscular mycorrhizal fungal communities on restoration success. Funct Ecol 35:2621–2634. doi:10.1111/1365-2435.13914

[14] Färkkilä SMA, Valtonen A, Saravesi K, Anslan S, Markkola A, Kontunen-Soppela S. 2023. The effects of geographic origin and genotype on fungal diversity of silver birch (Betula pendula). Fungal Ecol 63:101241. doi:10.1016/j.funeco.2023.101241

[15] Fierer N, Jackson RB. 2006. The diversity and biogeography of soil bacterial communities. Proc Natl Acad Sci U S A 103:626–631. doi:10.1073/pnas.0507535103 16407148 PMC1334650

[16] Keenan TF, Niinemets Ü. 2017. Global leaf trait estimates biased due to plasticity in the shade. Nat Plants 3:16201. doi:10.1038/nplants.2016.201 27991884

[17] Fry EL, Savage J, Hall AL, Oakley S, Pritchard WJ, Ostle NJ, Pywell RF, Bullock JM, Bardgett RD. 2018. Soil multifunctionality and drought resistance are determined by plant structural traits in restoring grassland. Ecology 99:2260–2271. doi:10.1002/ecy.2437 30129182 PMC6849565

[18] Bulgarelli D, Schlaeppi K, Spaepen S, Ver Loren van Themaat E, Schulze-Lefert P. 2013. Structure and functions of the bacterial microbiota of plants. Annu Rev Plant Biol 64:807–838. doi:10.1146/annurev-arplant-050312-120106 23373698

[19] Li Y, Wu X, Chen T, Wang W, Liu G, Zhang W, Li S, Wang M, Zhao C, Zhou H, Zhang G. 2018. Plant phenotypic traits eventually shape its microbiota: a common garden test. Front Microbiol 9:2479. doi:10.3389/fmicb.2018.02479 30459725 PMC6232875

[20] Mu D, Chen L, Hua G, Pu L, Tian Z, Liu Y, Zhang G, Tang J. 2023. Diversity and recruitment strategies of rhizosphere microbial communities by Camellia fascicularis, a plant species with extremely small populations in China: plant recruits special microorganisms to get benefit out of them. Diversity (Basel) 15:1170. doi:10.3390/d15121170

[21] Lamit LJ, Holeski LM, Flores-Rentería L, Whitham TG, Gehring CA. 2016. Tree genotype influences ectomycorrhizal fungal community structure: ecological and evolutionary implications. Fungal Ecol 24:124–134. doi:10.1016/j.funeco.2016.05.013

[22] Schechter SP, Bruns TD. 2008. Serpentine and non-serpentine ecotypes of Collinsia sparsiflora associate with distinct arbuscular mycorrhizal fungal assemblages. Mol Ecol 17:3198–3210. doi:10.1111/j.1365-294X.2008.03828.x 18611218

[23] Unuk T, Martinović T, Finžgar D, Šibanc N, Grebenc T, Kraigher H. 2019. Root-associated fungal communities from two phenologically contrasting silver fir (Abies alba mill.) groups of trees. Front Plant Sci 10:214. doi:10.3389/fpls.2019.00214 30891052 PMC6413537

[24] Gallart M, Adair KL, Love J, Meason DF, Clinton PW, Xue J, Turnbull MH. 2018. Host genotype and nitrogen form shape the root microbiome of Pinus radiata. Microb Ecol 75:419–433. doi:10.1007/s00248-017-1055-2 28875273

[25] Gehring CA, Sthultz CM, Flores-Rentería L, Whipple AV, Whitham TG. 2017. Tree genetics defines fungal partner communities that may confer drought tolerance. Proc Natl Acad Sci U S A 114:11169–11174. doi:10.1073/pnas.1704022114 28973879 PMC5651740

[26] Treseder KK, Vitousek PM. 2001. Potential ecosystem-level effects of genetic variation among populations of Metrosideros polymorpha from a soil fertility gradient in Hawaii. Oecologia 126:266–275. doi:10.1007/s004420000523 28547626

[27] Schweitzer JA, Bailey JK, Rehill BJ, Martinsen GD, Hart SC, Lindroth RL, Keim P, Whitham TG. 2004. Genetically based trait in a dominant tree affects ecosystem processes. Ecol Lett 7:127–134. doi:10.1111/j.1461-0248.2003.00562.x

[28] Madritch MD, Lindroth RL. 2011. Soil microbial communities adapt to genetic variation in leaf litter inputs. Oikos 120:1696–1704. doi:10.1111/j.1600-0706.2011.19195.x

[29] Campisano A, Albanese D, Yousaf S, Pancher M, Donati C, Pertot I. 2017. Temperature drives the assembly of endophytic communities' seasonal succession. Environ Microbiol 19:3353–3364. doi:10.1111/1462-2920.13843 28654220

[30] Faticov M, Ekholm A, Roslin T, Tack AJM. 2020. Climate and host genotype jointly shape tree phenology, disease levels and insect attacks. Oikos 129:391–401. doi:10.1111/oik.06707

[31] Cox SE, Stushnoff C. 2001. Temperature-related shifts in soluble carbohydrate content during dormancy and cold acclimation in Populus tremuloides. Can J For Res 31:730–737. doi:10.1139/x00-206

[32] Li HM, Sullivan R, Moy M, Kobayashi DY, Belanger FC. 2004. Expression of a novel chitinase by the fungal endophyte in Poa ampla. Mycologia 96:526–536. doi:10.1080/15572536.2005.11832951 21148875

[33] Renaut J, Hoffmann L, Hausman J-F. 2005. Biochemical and physiological mechanisms related to cold acclimation and enhanced freezing tolerance in poplar plantlets. Physiol Plant 125:82–94. doi:10.1111/j.1399-3054.2005.00554.x

[34] Jansson S, Douglas CJ. 2007. Populus: a model system for plant biology. Annu Rev Plant Biol 58:435–458. doi:10.1146/annurev.arplant.58.032806.103956 17280524

[35] Shen SY, Fulthorpe R. 2015. Seasonal variation of bacterial endophytes in urban trees. Front Microbiol 6:427. doi:10.3389/fmicb.2015.00427 26042095 PMC4437045

[36] Barberán A, Bates ST, Casamayor EO, Fierer N. 2012. Using network analysis to explore co-occurrence patterns in soil microbial communities. ISME J 6:343–351. doi:10.1038/ismej.2011.119 21900968 PMC3260507

[37] van der Heijden MGA, Bardgett RD, van Straalen NM. 2008. The unseen majority: soil microbes as drivers of plant diversity and productivity in terrestrial ecosystems. Ecol Lett 11:296–310. doi:10.1111/j.1461-0248.2007.01139.x 18047587

[38] Deng Y, Jiang YH, Yang YF, He ZL, Luo F, Zhou JZ. 2012. Molecular ecological network analyses. BMC Bioinformatics 13:113. doi:10.1186/1471-2105-13-113 22646978 PMC3428680

[39] Hartman K, van der Heijden MGA, Wittwer RA, Banerjee S, Walser J-C, Schlaeppi K. 2018. Cropping practices manipulate abundance patterns of root and soil microbiome members paving the way to smart farming. Microbiome 6:14. doi:10.1186/s40168-017-0389-9 29338764 PMC5771023

[40] Furtado BU, Gołębiewski M, Skorupa M, Hulisz P, Hrynkiewicz K. 2019. Bacterial and fungal endophytic microbiomes of Salicornia europaea. Appl Environ Microbiol 85:e00305-19. doi:10.1128/AEM.00305-19 31003988 PMC6581177

[41] Thiem D, Goebel M, Gołębiewski M, Baum C, Koczorski P, Szymańska S, Hrynkiewicz K. 2023. Endophytic microbiota and ectomycorrhizal structure of Alnus glutinosa Gaertn. at saline and nonsaline forest sites. Sci Rep 13:22831. doi:10.1038/s41598-023-49447-w 38129474 PMC10739818

[42] Agler MT, Ruhe J, Kroll S, Morhenn C, Kim ST, Weigel D, Kemen EM. 2016. Microbial hub taxa link host and abiotic factors to plant microbiome variation. PLoS Biol 14:e1002352. doi:10.1371/journal.pbio.1002352 26788878 PMC4720289

[43] Li J, Liu Y-X, Lü P-P, Wang Y-L, Li Z-F, Zhang Y, Gan H-Y, Li X-C, Mandal D, Cai J, Guo Z-X, Yao H, Guo L-D. 2022. Community assembly of fungi and bacteria along soil-plant continuum differs in a Zoige wetland. Microbiol Spectr 10:e0226022. doi:10.1128/spectrum.02260-22 36135597 PMC9604091

[44] Hernández-Terán A, Navarro-Díaz M, Benítez M, Lira R, Wegier A, Escalante AE. 2020. Host genotype explains rhizospheric microbial community composition: the case of wild cotton metapopulations (Gossypium hirsutum L.) in Mexico. FEMS Microbiol Ecol 96:fiaa109. doi:10.1093/femsec/fiaa109 32490512

[45] Bowsher AW, Benucci GMN, Bonito G, Shade A. 2021. Seasonal dynamics of core fungi in the switchgrass phyllosphere, and co-occurrence with leaf bacteria. Phytobiomes J 5:60–68. doi:10.1094/PBIOMES-07-20-0051-R

[46] Boratyński A. 1993. Systematics and geographical distribution, p 112–125. In Białobok S, Boratyński K, Bugała W (ed), Biology of Scots pine. Sorus, Poznań–Kórnik.

[47] Giertych M, Oleksyn J. 1992. Studies on genetic variation in Scots pine (Pinus sylvestris L.) coordinated by IUFRO. Silvae Genet 41:133–143.

[48] Zadworny M, McCormack ML, Mucha J, Reich PB, Oleksyn J. 2016. Scots pine fine roots adjust along a 2000-km latitudinal climatic gradient. New Phytol 212:389–399. doi:10.1111/nph.14048 27301778

[49] Karger DN, Conrad O, Böhner J, Kawohl T, Kreft H, Soria-Auza RW, Zimmermann NE, Linder HP, Kessler M. 2017. Climatologies at high resolution for the earth’s land surface areas. Sci Data 4:170122. doi:10.1038/sdata.2017.122 28872642 PMC5584396

[50] Oleksyn J, Zytkowiak R, Karolewski P, Reich PB, Tjoelker MG. 2000. Genetic and environmental control of seasonal carbohydrate dynamics in trees of diverse Pinus sylvestris populations. Tree Physiol 20:837–847. doi:10.1093/treephys/20.12.837 12651505

[51] Caporaso JG, Kuczynski J, Stombaugh J, Bittinger K, Bushman FD, Costello EK, Fierer N, Peña AG, Goodrich JK, Gordon JI, et al.. 2010. QIIME allows analysis of high-throughput community sequencing data. Nat Methods 7:335–336. doi:10.1038/nmeth.f.303 20383131 PMC3156573

[52] Bokulich NA, Subramanian S, Faith JJ, Gevers D, Gordon JI, Knight R, Mills DA, Caporaso JG. 2013. Quality-filtering vastly improves diversity estimates from Illumina amplicon sequencing. Nat Methods 10:57–59. doi:10.1038/nmeth.2276 23202435 PMC3531572

[53] Kõljalg U, Nilsson RH, Abarenkov K, Tedersoo L, Taylor AFS, Bahram M, Bates ST, Bruns TD, Bengtsson-Palme J, Callaghan TM, et al.. 2013. Towards a unified paradigm for sequence-based identification of fungi. Mol Ecol 22:5271–5277. doi:10.1111/mec.12481 24112409

[54] Kisuse J, La-Ongkham O, Nakphaichit M, Therdtatha P, Momoda R, Tanaka M, Fukuda S, Popluechai S, Kespechara K, Sonomoto K, Lee Y-K, Nitisinprasert S, Nakayama J. 2018. Urban diets linked to gut microbiome and metabolome alterations in children: a comparative cross-sectional study in Thailand. Front Microbiol 9:1345. doi:10.3389/fmicb.2018.01345 29988433 PMC6024022

[55] Edgar RC, Haas BJ, Clemente JC, Quince C, Knight R. 2011. UCHIME improves sensitivity and speed of chimera detection. Bioinformatics 27:2194–2200. doi:10.1093/bioinformatics/btr381 21700674 PMC3150044

[56] Haas BJ, Gevers D, Earl AM, Feldgarden M, Ward DV, Giannoukos G, Ciulla D, Tabbaa D, Highlander SK, Sodergren E, Methé B, DeSantis TZ, Petrosino JF, Knight R, Birren BW, Human Microbiome Consortium. 2011. Chimeric 16S rRNA sequence formation and detection in Sanger and 454-pyrosequenced PCR amplicons. Genome Res 21:494–504. doi:10.1101/gr.112730.110 21212162 PMC3044863

[57] Edgar RC. 2013. UPARSE: highly accurate OTU sequences from microbial amplicon reads. Nat Methods 10:996–998. doi:10.1038/nmeth.2604 23955772

[58] Edgar RC. 2016. SINTAX: a simple non-Bayesian taxonomy classifier for 16S and ITS sequences. bioRxiv. doi:10.1101/074161

[59] Peay KG, Russo SE, McGuire KL, Lim Z, Chan JP, Tan S, Davies SJ. 2015. Lack of host specificity leads to independent assortment of dipterocarps and ectomycorrhizal fungi across a soil fertility gradient. Ecol Lett 18:807–816. doi:10.1111/ele.12459 26032408

[60] R Development Core Team. 2014. R: a language and environment for statistical computing. R Foundation for Statistical Computing, Vienna, Austria. https://www.R-project.org.

[61] Pohlert T. 2014. The pairwise multiple comparison of mean ranks package (PMCMR). R package version 4.1. http://cran.r-project.org/package=PMCMR.

[62] Kolde R. 2015. pheatmap: pretty heatmaps. R package version 1.0.8. http://cran.r-project.org/package=pheatmap.

[63] Roberts DW. 2016. Labdsv: ordination and multivariate analysis for ecology. R package version 1.8-0. http://cran.r-project.org/package=labdsv.

[64] Clarke KR, Somerfield PJ, Chapman MG. 2006. On resemblance measures for ecological studies, including taxonomic dissimilarities and a zero-adjusted Bray–Curtis coefficient for denuded assemblages. J Exp Mar Biol Ecol 330:55–80. doi:10.1016/j.jembe.2005.12.017

[65] Oksanen J, Blanchet FG, Kindt R, Legendre P, Minchin PR, O’Hara RB, Simpson GL, Solymos P, Stevens MHH, Wagner H. 2013. Vegan: community ecology package. R package version 2.0-7. http://CRAN.R-project.org/package=vegan.

[66] Abbas-Aghababazadeh F, Li Q, Fridley BL. 2018. Comparison of normalization approaches for gene expression studies completed with high-throughput sequencing. PLoS One 13:e0206312. doi:10.1371/journal.pone.0206312 30379879 PMC6209231

[67] Csardi G, Nepusz T. 2006. The igraph software package for complex network research. Inter J Complex Systems 1695. https://www.researchgate.net/publication/221995787_The_Igraph_Software_Package_for_Complex_Network_Research.

[68] Clauset A, Newman MEJ, Moore C. 2004. Finding community structure in very large networks. Phys Rev E 70. doi:10.1103/PhysRevE.70.066111 15697438

[69] Scheffer M, Carpenter SR, Lenton TM, Bascompte J, Brock W, Dakos V, van de Koppel J, van de Leemput IA, Levin SA, van Nes EH, Pascual M, Vandermeer J. 2012. Anticipating critical transitions. Science 338:344–348. doi:10.1126/science.1225244 23087241

[70] Terhonen E, Blumenstein K, Kovalchuk A, Asiegbu FO. 2019. Forest tree microbiomes and associated fungal endophytes: functional roles and impact on forest health. Fores 10:42. doi:10.3390/f10010042

[71] Leopold DR, Busby PE. 2020. Host genotype and colonist arrival order jointly govern plant microbiome composition and function. Curr Biol 30:3260–3266. doi:10.1016/j.cub.2020.06.011 32679100

[72] Isaac-Renton M, Montwé D, Hamann A, Spiecker H, Cherubini P, Treydte K. 2018. Northern forest tree populations are physiologically maladapted to drought. Nat Commun 9:5254. doi:10.1038/s41467-018-07701-0 30531998 PMC6288165

[73] Ramírez-Valiente JA, Deacon NJ, Etterson J, Center A, Sparks JP, Sparks KL, Longwell T, Pilz G, Cavender-Bares J. 2018. Natural selection and neutral evolutionary processes contribute to genetic divergence in leaf traits across a precipitation gradient in the tropical oak Quercus oleoides. Mol Ecol 27:2176–2192. doi:10.1111/mec.14566 29577469

[74] Des Roches S, Post DM, Turley NE, Bailey JK, Hendry AP, Kinnison MT, Schweitzer JA, Palkovacs EP. 2018. The ecological importance of intraspecific variation. Nat Ecol Evol 2:57–64. doi:10.1038/s41559-017-0402-5 29203921

[75] Chauvin T, Cochard H, Segura V, Rozenberg P. 2019. Native-source climate determines the Douglas-fir potential of adaptation to drought. For Ecol Manage 444:9–20. doi:10.1016/j.foreco.2019.03.054

[76] Roskilly B, Keeling E, Hood S, Giuggiola A, Sala A. 2019. Conflicting functional effects of xylem pit structure relate to the growth-longevity trade-off in a conifer species. Proc Natl Acad Sci U S A 116:15282–15287. doi:10.1073/pnas.1900734116 31209057 PMC6660759

[77] Wagner MR, Lundberg DS, Del Rio TG, Tringe SG, Dangl JL, Mitchell-Olds T. 2016. Host genotype and age shape the leaf and root microbiomes of a wild perennial plant. Nat Commun 7:12151. doi:10.1038/ncomms12151 27402057 PMC4945892

[78] Abrego N, Crosier B, Somervuo P, Ivanova N, Abrahamyan A, Abdi A, Hämäläinen K, Junninen K, Maunula M, Purhonen J, Ovaskainen O. 2020. Fungal communities decline with urbanization—more in air than in soil. ISME J 14:2806–2815. doi:10.1038/s41396-020-0732-1 32759974 PMC7784924

[79] Whipps JM, Hand P, Pink D, Bending GD. 2008. Phyllosphere microbiology with special reference to diversity and plant genotype. J Appl Microbiol 105:1744–1755. doi:10.1111/j.1365-2672.2008.03906.x 19120625

[80] Redford AJ, Bowers RM, Knight R, Linhart Y, Fierer N. 2010. The ecology of the phyllosphere: geographic and phylogenetic variability in the distribution of bacteria on tree leaves. Environ Microbiol 12:2885–2893. doi:10.1111/j.1462-2920.2010.02258.x 20545741 PMC3156554

[81] Bodenhausen N, Bortfeld-Miller M, Ackermann M, Vorholt JA. 2014. A synthetic community approach reveals plant genotypes affecting the phyllosphere microbiota. PLoS Genet 10:e1004283. doi:10.1371/journal.pgen.1004283 24743269 PMC3990490

[82] Bogino P, Abod A, Nievas F, Giordano W. 2013. Water-limiting conditions alter the structure and biofilm-forming ability of bacterial multispecies communities in the alfalfa rhizosphere. PLoS One 8:e79614. doi:10.1371/journal.pone.0079614 24223979 PMC3817132

[83] Maignien L, DeForce EA, Chafee ME, Eren AM, Simmons SL. 2014. Ecological succession and stochastic variation in the assembly of Arabidopsis thaliana phyllosphere communities. mBio 5:e00682-13. doi:10.1128/mBio.00682-13 24449749 PMC3903271

[84] Müller DB, Vogel C, Bai Y, Vorholt JA. 2016. The plant microbiota: systems-level insights and perspectives. Annu Rev Genet 50:211–234. doi:10.1146/annurev-genet-120215-034952 27648643

[85] Wachowiak W, Perry A, Zaborowska J, González-Martínez SC, Cavers S. 2022. Admixture and selection patterns across the European distribution of Scots pine, Pinus sylvestris (Pinaceae). Bot J Linnean Soc 200:416–432. doi:10.1093/botlinnean/boac016

[86] Gao C, Xu L, Montoya L, Madera M, Hollingsworth J, Chen L, Purdom E, Singan V, Vogel J, Hutmacher RB, Dahlberg JA, Coleman-Derr D, Lemaux PG, Taylor JW. 2022. Co-occurrence networks reveal more complexity than community composition in resistance and resilience of microbial communities. Nat Commun 13:3867. doi:10.1038/s41467-022-31343-y 35790741 PMC9256619

[87] Newman MEJ. 2006. Modularity and community structure in networks. Proc Natl Acad Sci U S A 103:8577–8582. doi:10.1073/pnas.0601602103 16723398 PMC1482622

[88] Krause AE, Frank KA, Mason DM, Ulanowicz RE, Taylor WW. 2003. Compartments revealed in food-web structure. Nat New Biol 426:282–285. doi:10.1038/nature02115 14628050

[89] Gan H, Li X, Wang Y, Lü P, Ji N, Yao H, Li S, Guo L. 2022. Plants play stronger effects on soil fungal than bacterial communities and co-occurrence network structures in a subtropical tree diversity experiment. Microbiol Spectr 10:e0013422. doi:10.1128/spectrum.00134-22 35475656 PMC9241759

