# Supplementary material for: The effects of Pinus sylvestris L. geographical origin on the community and co-occurrence of fungal and bacterial endophytes in a common garden experiment
Source: Microbiol Spectr. 2024 Sep 9;12(10):e00807-24. doi: 10.1128/spectrum.00807-24 (PMC11448405; doi:10.1128/spectrum.00807-24)
Supplement: Supplemental material — Fig. S1 to S5; Tables S1 and S2, S5. [file spectrum.00807-24-s0001.docx]

**SUPLEMENTARY MATERIALS**


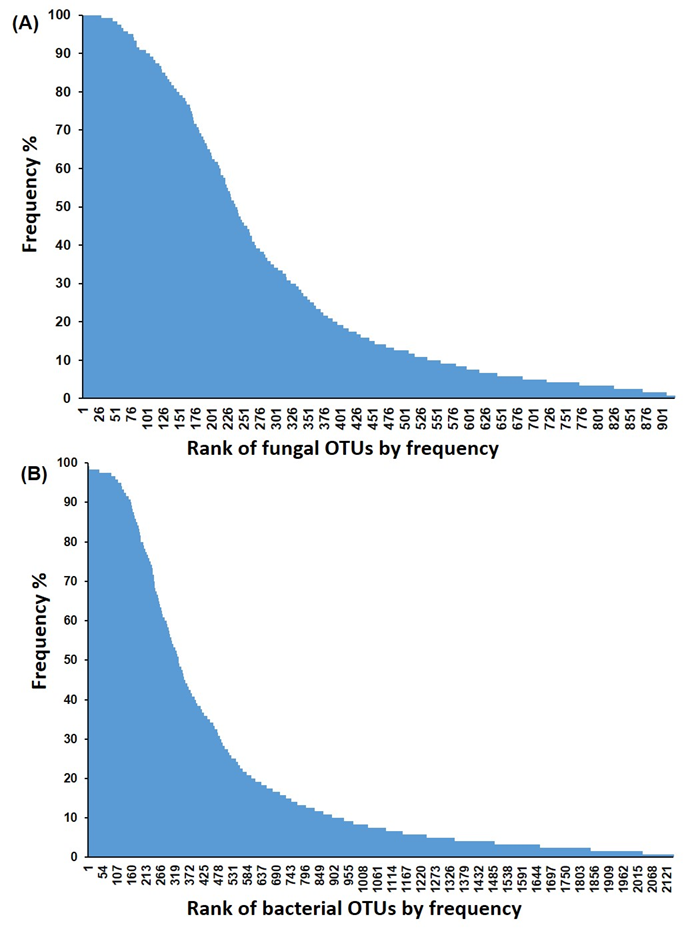


**FIG S1** Rank of fungal (A) and bacterial (B) OTUs by frequency


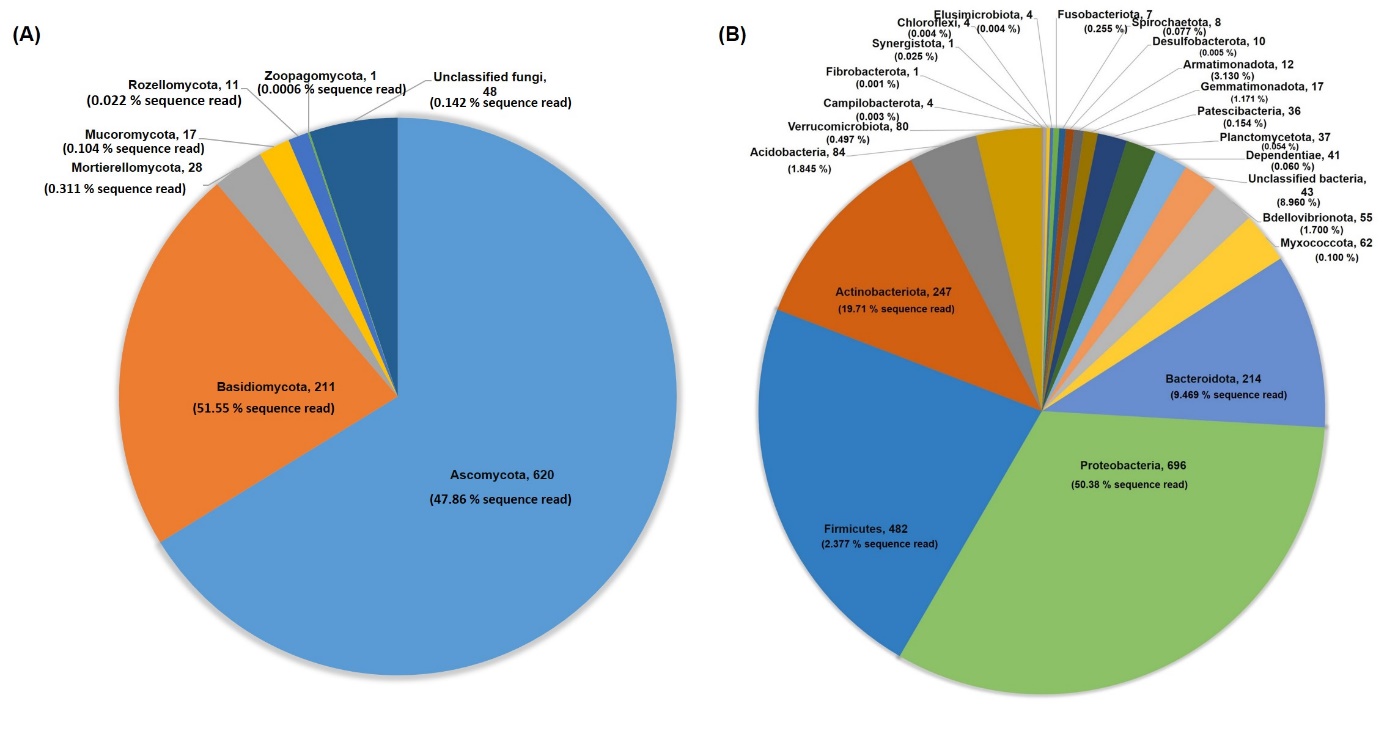


**FIG S2** Number of OTUs in different fungal (A) and bacterial (B) phylum (% of sequence abundance indicated in the parenthesis)


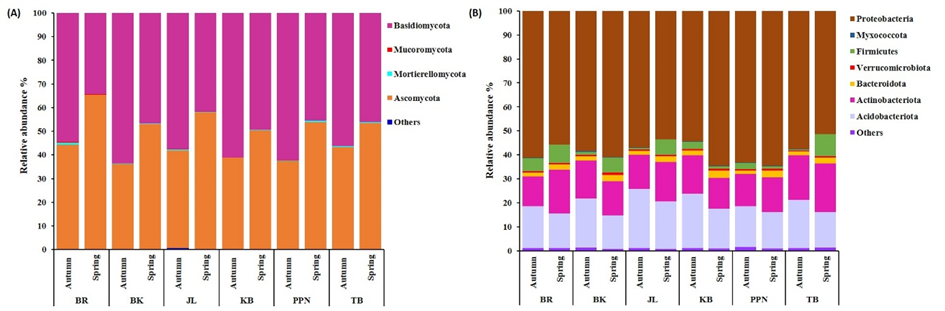


**FIG S3** Relative abundance of fungal and bacterial endophytes at the phylum level in different tree origin of *P. sylvestris* in two growing seasons. The fungal and bacterial phylum represents < 0.1% of the total reads and fungi and bacteria which are not identified to phylum level were all assigned to “Others”.


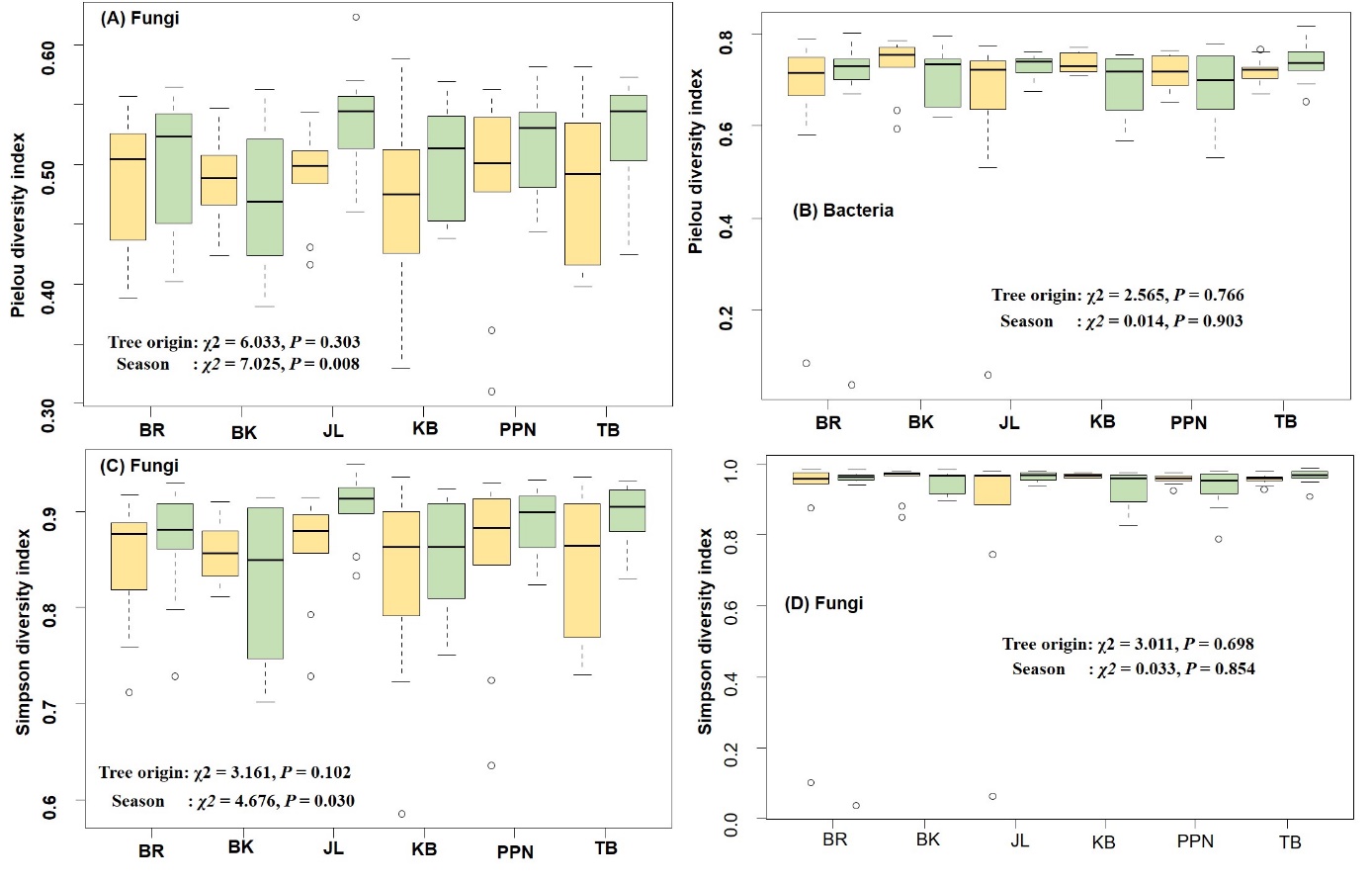


**FIG S4** Pielou (**A** and **B**) and Simpson (**A** and **B**) diversity index of fungal and bacterial endophytes in different tree origin of *P. sylvestris*. The black line inside each box represents the median value (n= 10). Kruskal–Wallis test revealed the effect of tree origin and season on the OTU richness and Shannon diversity index of endophytic fungi and bacteria.


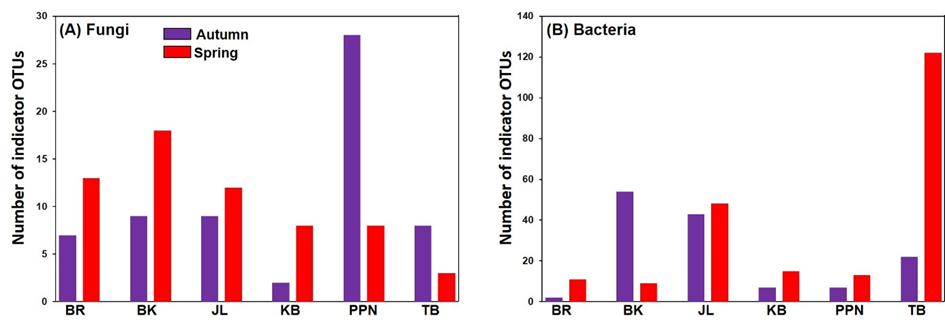


**FIG S5** Distribution of indicator OTUs of endophytic fungi (**A**) and bacteria (**B**) in different tree origin of *P. sylvestris* in different season as revealed by indicator species analysis of the relative abundance of endophytic fungal and bacterial OTUs

| **TABLE S1** Climatic variables of the geographical origin of *P. sylvestris* | | | | | | |
| --- | --- | --- | --- | --- | --- | --- |
| Climatic variables | Geographical origin | | | | | |
|  | PPN | BK | JL | BR | KB | TB |
| Longitude | 19.33 | 16.6 | 22.42 | 14.83 | 17.09 | 20 |
| Latitude | 49.33 | 50.28 | 50.67 | 51.77 | 52.25 | 53.57 |
| Annual Mean Temperature (°C) | 5.97 | 7.18 | 8.38 | 9.5 | 9 | 7.28 |
| Max Temperature of Warmest Month (°C) | 20.54 | 21.78 | 23.38 | 23.6 | 23.54 | 21.88 |
| Min Temperature of Coldest Month (°C) | -7.38 | -5.72 | -5 | -2.3 | -3.06 | -5.07 |
| Temperature Annual Range | 27.95 | 27.5 | 28.38 | 25.93 | 26.6 | 27 |
| Mean Temperature of Wettest Quarter (°C) | 15.44 | 16.48 | 18.3 | 19 | 18.35 | 16.85 |
| Mean Temperature of Driest Quarter (°C) | -3.63 | -2.28 | -1.9 | 3.2 | -0.3 | -2.37 |
| Mean Temperature of Warmest Quarter (°C) | 15.74 | 16.8 | 18.4 | 19 | 18.69 | 17.15 |
| Mean Temperature of Coldest Quarter (°C) | -3.93 | -2.37 | -1.9 | 0.5 | -0.3 | -2.37 |
| Annual Mean Precipitation ( mm) | 844 | 670 | 625 | 590 | 505 | 547 |
| Precipitation of Wettest Month (mm) | 108 | 86 | 87 | 68 | 74 | 73 |
| Precipitation of Driest Month (mm) | 43 | 35 | 30 | 35 | 25 | 25 |
| Precipitation Seasonality (mm) | 32 | 34 | 36 | 22 | 34 | 35 |
| Precipitation of Wettest Quarter (mm) | 321 | 258 | 255 | 202 | 208 | 218 |
| Precipitation of Driest Quarter | 131 | 109 | 91 | 109 | 78 | 78 |
| Precipitation of Warmest Quarter (mm) | 291 | 250 | 231 | 202 | 189 | 198 |
| Precipitation of Coldest Quarter (mm) | 147 | 124 | 91 | 136 | 78 | 78 |

| **TABLE S2** Root biochemical parameters of *P. sylvestris* originated from different geographical location in two season. | | | | | | |
| --- | --- | --- | --- | --- | --- | --- |
| Month | Origin | Glucose (%) | Starch (%) | TNC (%) | Carbon (%) | Nitrogen (%) |
| Autumn | BR | 4.059 ± 0.267 a | 0.859 ± 0.218 a | 4.919 ± 0.375 a | 48.66 ± 0.604 a | 1.639 ± 0.057 a |
|  | BK | 3.506 ± 0.174 a | 1.562 ± 0.426 a | 5.068 ± 0.438 a | 49.98 ± 0.457 a | 1.660 ± 0.051 a |
|  | JL | 3.405 ± 0.318 a | 1.692 ± 0.522 a | 5.097 ± 0.693 a | 49.84 ± 0.884 a | 1.672 ± 0.074 a |
|  | KB | 3.387 ± 0.260 a | 1.124 ± 0.210 a | 4.512 ± 0.383 a | 48.69 ± 0.807 a | 1.636 ± 0.031 a |
|  | PPN | 4.205 ± 0.674 a | 0.891 ± 0.167 a | 5.096 ± 0.713 a | 49.45 ± 0.871 a | 1.782 ± 0.059 a |
|  | TB | 4.115 ± 0.396 a | 1.521 ± 0.371 a | 5.637 ± 0.561 a | 48.53 ± 0.560 a | 1.606 ± 0.040 a |
| Spring | BR | 6.778 ± 1.271 a | 3.955 ± 0.652 ab | 10.73 ± 1.583 ab | 47.09 ± 0.921 a | 1.609 ± 0.072 a |
|  | BK | 4.724 ± 0.263 a | 3.754 ± 0.723 b | 8.478 ± 0.838 b | 47.14 ± 0.800 a | 1.698 ± 0.079 a |
|  | JL | 4.564 ± 0.341 a | 4.695 ± 0.717 ab | 9.260 ± 0.829 ab | 47.63 ± 0.380 a | 1.573 ± 0.071 a |
|  | KB | 4.802 ± 0.201 a | 4.884 ± 0.667 ab | 9.686 ± 0.740 ab | 46.93 ± 0.249 a | 1.513 ± 0.034 a |
|  | PPN | 5.833 ± 0.445 a | 6.926 ± 0.912 a | 12.76 ± 0.863 a | 47.63 ± 0.193 a | 1.587 ± 0.028 a |
|  | TB | 5.086 ± 0.334 a | 4.016 ± 0.720 ab | 9.102 ± 0.827 ab | 46.73 ± 0.339 a | 1.732 ± 0.082 a |
| Data (means ± SE, n = 10) in the same column with different letters are significantly different at *P* < 0.05 according to Tukey’s HSD test in each season. | | | | | | |

| **TABLE S5** Topological network properties of the co-occurrence network of fungal and bacterial endophytes in different grographical origin of *P. sylvestris* in two season. | | | |
| --- | --- | --- | --- |
| Seed origin | Season | Connectivity | Modularity |
| BR | autumn | 0.049287 | 0.693143 |
|  | spring | 0.031484 | 0.824157 |
| BK | autumn | 0.028904 | 0.858745 |
|  | spring | 0.035726 | 0.769236 |
| JL | autumn | 0.037162 | 0.87096 |
|  | spring | 0.028712 | 0.857166 |
| KB | autumn | 0.035121 | 0.829872 |
|  | spring | 0.038348 | 0.670609 |
| PPN | autumn | 0.033367 | 0.788548 |
|  | spring | 0.031814 | 0.754008 |
| TB | autumn | 0.03391 | 0.798837 |
|  | spring | 0.030995 | 0.832163 |
